# Graphene overcoats for ultra-high storage density magnetic media

**DOI:** 10.1038/s41467-021-22687-y

**Published:** 2021-05-17

**Authors:** N. Dwivedi, A. K. Ott, K. Sasikumar, C. Dou, R. J. Yeo, B. Narayanan, U. Sassi, D. De Fazio, G. Soavi, T. Dutta, O. Balci, S. Shinde, J. Zhang, A. K. Katiyar, P. S. Keatley, A. K. Srivastava, S. K. R. S. Sankaranarayanan, A. C. Ferrari, C. S. Bhatia

**Affiliations:** 1 CSIR-Advanced Materials and Processes Research Institute, Bhopal, India; 2 Department of Electrical and Computer Engineering, National University of Singapore, Singapore, Singapore; 3 Cambridge Graphene Centre, University of Cambridge, Cambridge, UK; 4 Department of Engineering, University of Exeter, Exeter, UK; 5 Center for Nanoscale Materials, Argonne National Laboratory, Argonne, IL USA; 6 Institute of Materials, Ecole Polytechnique Fédérale de Lausanne (EPFL), Lausanne, Switzerland; 7 Empa-Swiss Federal Laboratories for Material Science and Technology, Dübendorf, Switzerland; 8 Department of Physics and Astronomy, University of Exeter, Exeter, UK; 9 Department of Mechanical and Industrial Engineering, University of Illinois, Chicago, IL USA

**Keywords:** Materials science, Materials for devices, Nanoscale materials

## Abstract

Hard disk drives (HDDs) are used as secondary storage in digital electronic devices owing to low cost and large data storage capacity. Due to the exponentially increasing amount of data, there is a need to increase areal storage densities beyond ~1 Tb/in^2^. This requires the thickness of carbon overcoats (COCs) to be <2 nm. However, friction, wear, corrosion, and thermal stability are critical concerns below 2 nm, limiting current technology, and restricting COC integration with heat assisted magnetic recording technology (HAMR). Here we show that graphene-based overcoats can overcome all these limitations, and achieve two-fold reduction in friction and provide better corrosion and wear resistance than state-of-the-art COCs, while withstanding HAMR conditions. Thus, we expect that graphene overcoats may enable the development of 4–10 Tb/in^2^ areal density HDDs when employing suitable recording technologies, such as HAMR and HAMR+bit patterned media

## Abstract (web summary)

The main limitation to the areal storage density of hard disk drives (HDDs) is the thickness of carbon overcoats protecting the disk media. Here, <2-nm-thick graphene overcoats with improved corrosion, wear and irradiation resistance are shown to meet the requirements for the realization of 4–10 Tb/in^2^ HDDs with heat assisted magnetic recording.

## Introduction

There has been an incessant increase in data generation over the past few decades^1^. The annual data creation rate was ~16.1 zettabytes/year (ZB, 1ZB = trillion gigabytes (GB)) in 2016^1^, and is expected to increase to ~163 ZB/year by 2025^1^. While various devices are used to store digital information, e.g., tape^2^ and flash drives^3,4^, hard disk drives (HDDs) remain the primary choice as a secondary storage devices, due to their low cost <0.1$/GB at 2016 prices^3^ and large storage capacity >10 TB with 3.5 inch HDDs^3^. HDDs will rule storage technologies at least for the next 5–10 years^5^ in terms of capacity^3,4^, price^3,4^, production^3,4^, and shipment^3,4^. Supplementary Note 1 provides the key background concepts on HDD technology and the main technological challenges.

Solid state drives^3,4^ are the main competing technology, posing a threat to future HDD viability^4^. Thus, novel technologies are needed to enable HDDs with high areal density (AD) >1 Tb/in^2^^3,6^.

**Fig. 1:**
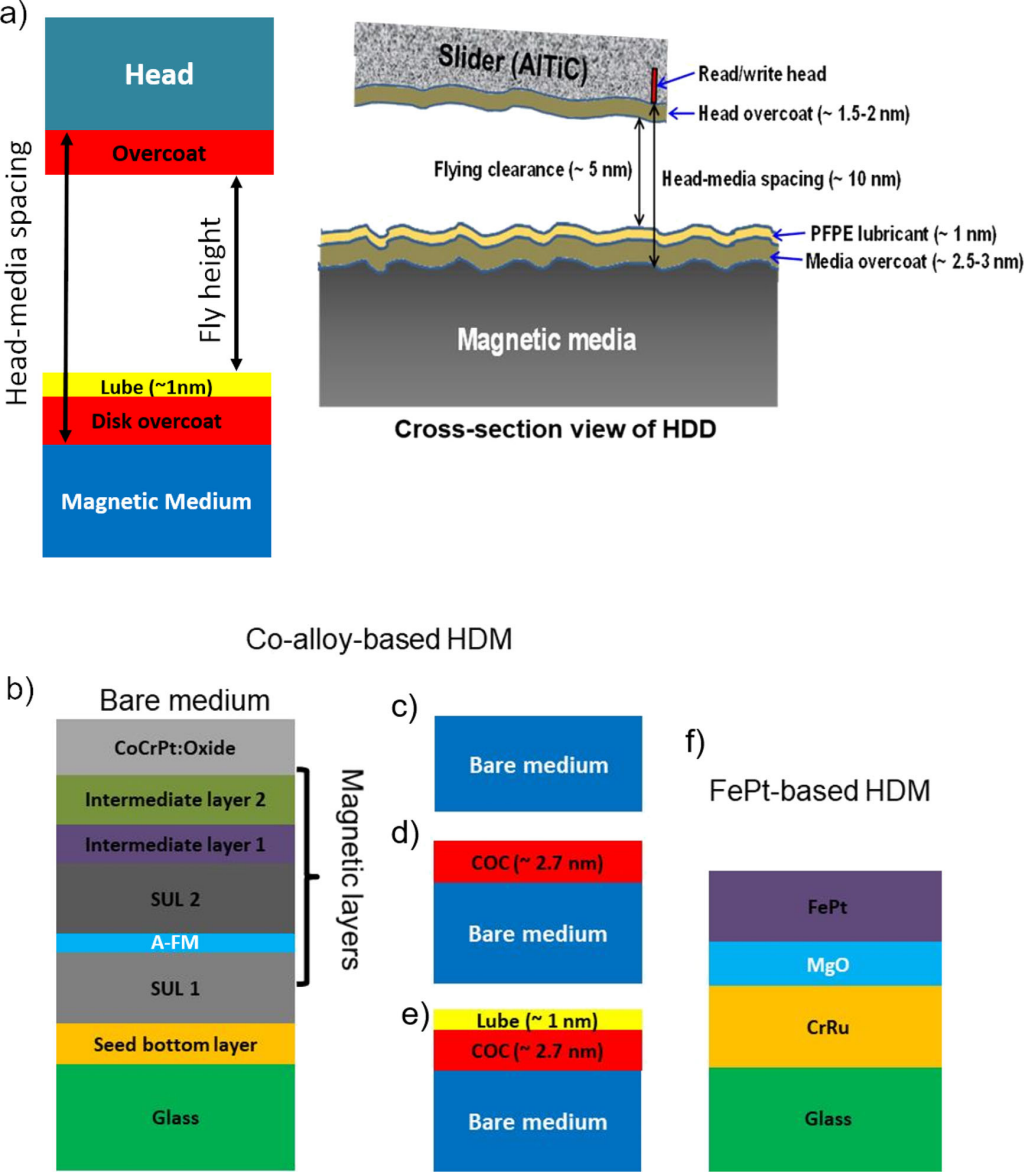
Hard disk media structures. **a** Schematic cross-section of a hard disk drive with magnetic medium, disk overcoat, lubricant, fly height, head overcoat, head. The head-medium spacing and fly height/clearance are indicated by arrows. The slider containing the read/write head and the head overcoat are shown as well. For 1 Tb/in^2^, the head-media spacing is ~8.9–6.5 nm^6^, based on the sum of overcoat thickness (2.5–2 nm)^6^, lubricant thickness (1.2–1 nm)^6^, touch down height (2–1 nm)^6^, fly clearance (1.2–1 nm)^6^ and head overcoat thickness (2–1.5 nm)^6^. **b** Full stack of bare CoCrPt:Oxide-based hard disk media comprising a glass substrate, seed bottom underlayer, and antiferromagnetic layer (A-FM) between two soft-magnetic under layers (SUL), followed by two intermediate layers and a co-based magnetic recording layer. **c** Bare medium (BM). **d** Magnetic medium with ~2.7 nm commercial COC. **e** Magnetic medium with ~2.7 nm commercial COC and ~1 nm commercial lube. **f** Full stack of bare FePt-based hard disk medium (HDM) containing glass substrate, a layer of CrRu followed by layers of MgO and FePt.

One option is to reduce the head-hard disk medium (HDM) spacing, since this decreases the signal-to-noise ratio^6^ and limits AD growth^6^. There are many contributors to the head-HDM spacing (Fig. 1a and Supplementary Note 1), whereby the HDM overcoat is the largest contributor.

Carbon-based overcoats (COCs) are widely used to protect HDM from mechanical damage^7–12^ and corrosion^8,13–15^, to ensure maintained functionality and durability^13,14^. By extrapolating Fig. 2 in ref. ^6^, a COC thickness <1 nm would be required for ~10 Tb/in^2^. However, current COCs lose most of their appealing properties, such as anti-friction^11,12,15^, wear resistance^11,12^, Young’s Modulus^7,8,13,16^, and corrosion protection^7,8,13,16^, when their thickness is below 2–3 nm.

The ideal overcoat needs to provide: (1) corrosion protection, requiring complete coverage^11,14^ without pinholes^13,14^; (2) coefficient of friction (COF), i.e. the ratio of frictional to normal force, below 0.3–0.5, lower than in 2.7 nm commercial COCs^11,15^ and wear resistance^11,12,14^, requiring better lubricating properties^11,12^, hardness and elasticity with Young’s modulus of at least ~400 GPa^7,14^; (3) lubricant compatibility^14^; (4) surface smoothness^16–18^, i.e., root mean square roughness ~0.2–0.3 nm^14^. Given the limitations to achieve most of these properties with conventional COCs below 2 nm^7,8,13,19^, either the search for novel overcoats or engineering of existing overcoats is required to enable future ultra-high AD HDDs.

Another critical bottleneck hindering AD growth is the superparamagnetic limit^20,21^, which contributes to the magnetic trilemma, linking the three problems: AD increase, thermal stability, writability^6,20–22^ (see Supplementary Note 1).

To overcome writability issues in materials with high magnetic anisotropy, heat assisted magnetic recording (HAMR) was suggested^23–25^. HAMR uses a laser to heat the magnetic medium for ~1 ns^23^ in order to decrease its coercivity, bringing the material to its Curie temperature, *T*_C_, above which it is writable with a magnetic field ~0.8 T by conventional heads^26^. After cooling to room temperature, the coercivity goes back to its original value, and data is retained^27^. While HAMR appears to be a solution to all issues, such as small grain size, high magnetic anisotropy, and writability, and paves the way to increasing AD, it raises concerns on the thermal stability of COCs against laser irradiation^28–30^. As *T*_C_ is ~ 700–750 K for FePt^22,28,29^, reached by laser irradiation, the overcoats for HAMR require thermal stability at least up to ~700–800 K to avoid degradation over time^23,28,29^.

Efforts are ongoing to test the thermal stability of HDM overcoats, e.g., by using laser^28,29^ and thermal annealing^30^, and by changing the thermal treatment time^28,30,31^ and heating rate^28–31^, to design HAMR-compatible overcoats. Reference ^28^ reported oxidation, degradation, and removal of a ~5 nm hydrogenated amorphous carbon, a-C:H,-based commercial COC on HAMR-compatible HDM in HAMR-like conditions, with 0.25 s total irradiation time. Reference ^29^ also reported degradation of 4 nm a-C:H-based commercial COC on a FePt-based HDM in HAMR-like conditions, with total heating time ~0.1 ms, corresponding to a 5-year drive life or 157.68 × 10^6^ s^29^ (see Supplementary Note 1).

Better thermal stability was reported in filtered cathodic vacuum arc (FCVA)-based COCs under laser irradiation in HAMR-like conditions^29^ and thermal annealing up to ~940 K^32^, consistent with the good thermal stability, i.e., no change in *sp*^3^ content up to 1100 °C, found in tetrahedral a-C (ta-C) films^33^. However, FCVA-based COCs are not yet used as HDM overcoats due to the presence of macro-particles^6,34^.

Graphene is an emerging material for lubrication^35–39^, as well as oxidation^40^ and corrosion protection^41–43^. Reference ^37^ reported that single-layer graphene (1LG) reduced the steel COF from 0.9 to 0.3 with a coating lifetime up to 6500 cycles, and decreased the wear rate by two orders of magnitude. Multilayer graphene (MLG) (3–4 layers) showed excellent tribological performance with a COF < 0.2, a decrease of wear rate by three orders of magnitude, and sliding lifetime up to 47000 cycles on steel^37^. Reference ^35^ reported that 1LG exhibits superlubricity^35^ and reduced the COF by 2–3 times and wear rate by two orders of magnitude for Au-based electrical contacts^38^. 1LG decreases the oxidation and corrosion of various metals, such as Ni^40,41,43^, Co^41^, Fe^41^, Pt^41^, Cu^40,42,43^, Ag^42^. Reduced graphene oxide was also used as a barrier coating^44^. Reference ^45^ reported that suspended 1LG has good thermal stability up to 2600 K, with a thermal conductivity up ~ 2000 W/mK^46^. In ref. ^47^, we showed that it is possible to achieve high mobilities ~30,000 cm^2^ V/s at room temperature in wet transferred, polycrystalline 1LG. Thus, scalable processes, such as wet transfer, can be used for integration and packaging^47,48^. All these characteristics make 1LG promising as a protective overcoat for both existing and HAMR-based technologies.

Here we use 1–4 layers of chemical vapor deposition (CVD) grown graphene (1–4LG) transferred on Co-alloy (current technology) and FePt-based (HAMR technology) HDMs, and test friction, wear, corrosion, thermal stability, and lube compatibility. We demonstrate that 1LG shows better performances than current 2.7 nm COCs on Co-alloy-based HDM, and good tribological properties with a COF < 0.2 for >10,000 cycles. We achieve very low COF ~ 0.15 for 1LG, indicating high wear resistance and corrosion protection, with corrosion current densities < 5 nA/cm^2^ for 2–3LG. Thermal stability tests confirm that 1LG on FePt can withstand HAMR-like conditions, without degradation. Graphene’s superior performance and its thinness can enable the development of ultra-high-density magnetic data storage technologies, based either on current technology, as well as on HAMR, or HAMR combined with bit patterned media (BPM), where the magnetic storage layer is patterned into an array of pillars, each representing a single bit^49^. The combination of 1LG + HAMR + BPM may increase AD > 10 Tb/in^2^.

## Results

### Magnetic media substrates

We use CoCrPt:Oxide-based bare HDM (BM) from Hitachi Global Storage Technologies (now Western Digital). These comprise multiple layers: seed bottom layer, soft-magnetic under layers (SUL), antiferromagnetic layer (A-FM), intermediate layers, and CoCrPt:Oxide storage layer (Fig. 1a, b). We also use other HDM from Western Digital with ~2.7 nm commercial COC (CMC, Fig. 1c), and ~2.7 nm commercial COC + ~1 nm commercial PFPE lube (CMCL, Fig. 1d) as reference.

The details and nomenclature of the COCs are in Table 1. The structural properties of the FCVA and sputter-deposited COCs can be found in refs. ^50,51^. HAMR-compatible FePt-based HDMs are shown in Fig. 1f. See Supplementary Notes 2 and 3 for further details.

**Table 1:** List of samples.

BM	CoCrPt-oxide-based BM without COC and lube
BML	CoCrPt-oxide-based BM without COC, with PFPE lube (1.2 ± 0.2 nm)
CMC	CoCrPt-oxide-based commercial HDM+COC (~2.7 nm) without lube
CMCL	CoCrPt-oxide-based commercial HDM+COC (~2.7 nm)+PFPE lube (~1 nm)
1LG	1LG on CoCrPt-oxide-based commercial HDM
2LG	2LG on CoCrPt-oxide-based commercial HDM
3LG	3LG on CoCrPt-oxide-based commercial HDM
4LG	4LG on CoCrPt-oxide-based commercial HDM
1LGL	PFPE lube (1.2 ± 0.2)nm-coated 1LG on CoCrPt-oxide-based commercial HDM
2LGL	PFPE lube (1.2 ± 0.2)nm-coated 2LG on CoCrPt-oxide-based commercial HDM
3LGL	PFPE lube (1.2 ± 0.2)nm-coated 3LG on CoCrPt-oxide-based commercial HDM
4LGL	PFPE lube (1.2 ± 0.2)nm-coated 4LG on CoCrPt-oxide-based commercial HDM
3CF	FCVA-deposited ta-C (~0.3 nm) on CoCrPt-oxide-based commercial HDM
6CF	FCVA-deposited ta-C (~0.6 nm) on CoCrPt-oxide-based commercial HDM
12CF	FCVA-deposited ta-C (~1.2 nm) on CoCrPt-oxide-based commercial HDM
18CF	FCVA-deposited ta-C (~1.8 nm) on CoCrPt-oxide-based commercial HDM
8CS	Pulsed DC sputtered *sp*^2^ rich carbon (~0.8 nm) on CoCrPt-oxide commercial HDM
12CS	Pulsed DC sputtered *sp*^2^ rich carbon (~1.2 nm) on CoCrPt-oxide commercial HDM
FePt	FePt/MgO/CrRu/glass samples grown by magnetron sputtering. CrRu and MgO seed layers are grown at 400 °C and 1.5 mTorr; FePt is grown at 600 °C and 3.5 mTorr as discussed in ref. ^22^.

Bare media, commercial media with an overcoat, graphene-coated media, FCVA, and sputtered carbon overcoats with and without lubricant (indicated by L), with details on sample structure and corresponding acronyms.

### Graphene growth and transfer

**Fig. 2:**
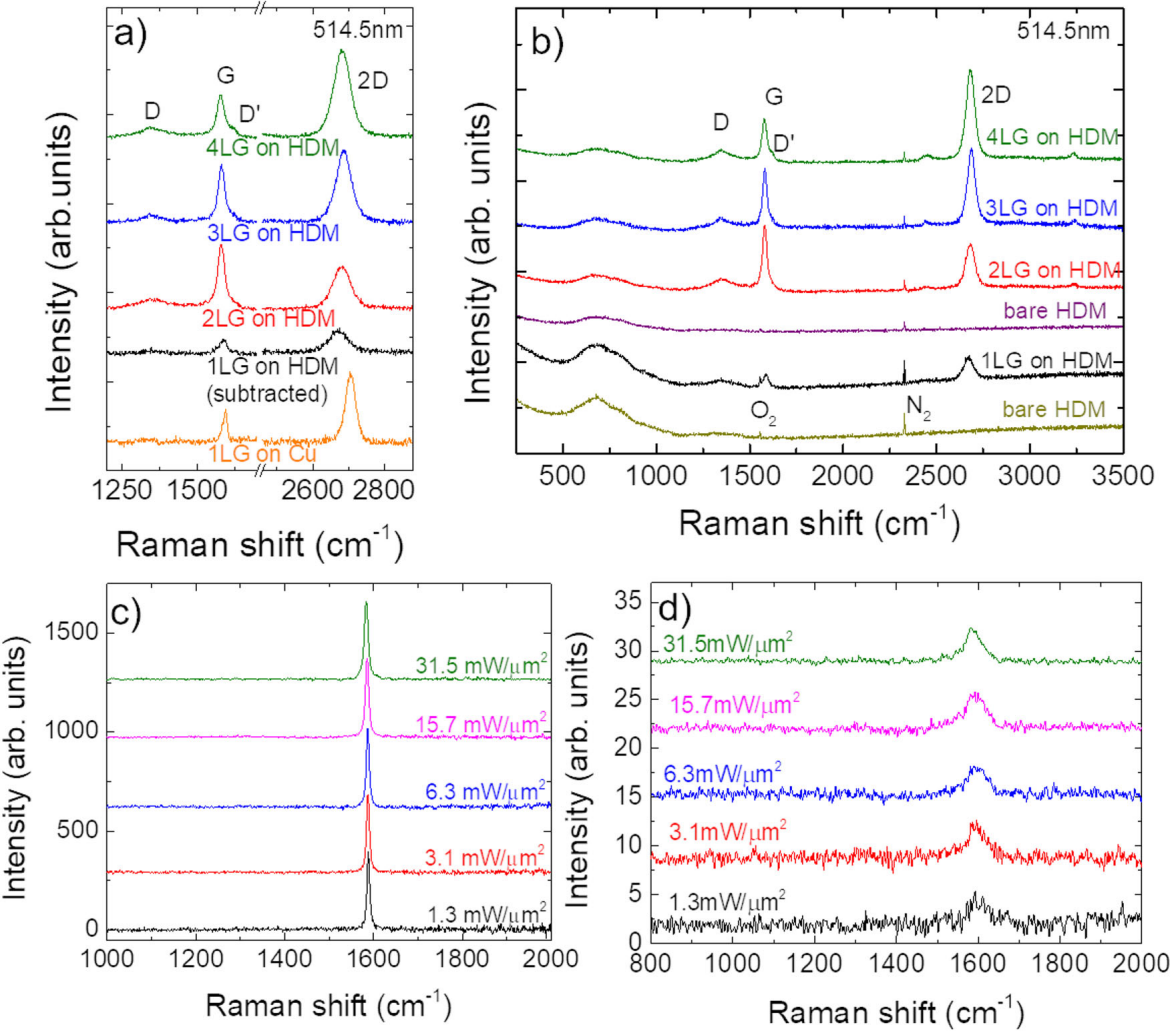
Raman and irradiation measurements. **a** 514.5 nm Raman spectra of as-grown 1LG on Cu (before transfer) and 1–4LG transferred on CoCrPt:Oxide after CoCrPt:Oxide background subtraction. **b** 514.5 nm Raman spectra of CoCrPt:Oxide substrates and 1–4LG transferred onto CoCrPt:Oxide. **c**, **d** 785 nm Raman spectra of 1LG on (**c**) Si/SiO_2_ and (**d**) FePt-based HAMR-HDM, for different laser power densities.

Graphene is grown by chemical vapor deposition (CVD) and placed on the HMD by wet transfer^52,53^, as described in the “Methods” section. The quality and uniformity of the samples are assessed by Raman spectroscopy^54^. Unpolarized Raman spectra are recorded at 514.5, as well as at 785 nm, close to the 850 nm used for HAMR writing tests^25,55^, with a Renishaw InVia spectrometer equipped with a Leica DM LM microscope and a ×100 objective with a numerical aperture 0.85. A 514.5 nm Raman spectrum of 1LG on Cu before the transfer is shown in Fig. 2a. The photoluminescence (PL) background due to the Cu foil is removed using baseline subtraction^56^. The D to G intensity ratio *I*(D)/*I*(G) is <0.1, indicating a defect concentration *n*_*d*_ < 2.4 × 10^10^ cm^−2^ ^54,57–59^ (Fig. 2b). The 2D peak position can be fitted with a single Lorentzian with Pos(2D) ~ 2705 cm^−1^ and full-width-half-maximum, FWHM(2D) ~ 33 cm^−1^, a signature of 1LG^54,60^. The G peak position and FWHM(G) are ~1593 cm^−1^ and 16 cm^−1^. *I*(2D)/*I*(G) and *A*(2D)/*A*(G) are ~2.3 and 4.9.

Representative Raman spectra of 1–4LG transferred on HDM are in Fig. 2a, with the HDM spectrum subtracted. The HDM spectrum and the spectra of 1L–4LG on HDM before background subtraction are in Fig. 2b. By subtracting the reference spectrum, using the N_2_ Raman peak from the air for normalization, from the spectrum taken on 1LG-coated HDM, we can reveal the 1LG contribution. After transfer, all peaks are downshifted and broader, with Pos(2D)_1LG_ ~ 2672 cm^−1^, Pos(2D)_2LG_ ~ 2681 cm^−1^, Pos(2D)_3LG_ ~ 2687 cm^−1^, Pos(2D)_4LG_ ~ 2682 cm^−1^, FWHM(2D)_1LG_ ~ 61 cm^−1^, FWHM(2D)_2LG_ ~ 57 cm^−1^, FWHM(2D)_3LG_ ~ 49 cm^−1^, FWHM(2D)_4LG_ ~ 53 cm^−1^. Assuming 2686 cm^−1^ as unstrained Pos(2D)^61^ and a rate of change in Pos(2D) with strain *δ*Pos(2D)/ *δϵ* ~ −64 cm^−1^/%^61^, this would give ~0.22% uniaxial strain in 1LG, consistent with the large FWHM(2D)^61,62^. We assume the strain to be mostly uniaxial, because it is unlikely to have biaxial, i.e., perfectly isotropic strain, in all directions, unless this is induced on purpose. There is a small increase in I(D)/I(G) to ~0.2 for 2–4LG on HDM.

Current commercial HDD technology uses a ~2.5–3 nm COC deposited by plasma-based processes^12,63^. The thickness and roughness of 1–4LG are measured by Atomic Force Microscopy (AFM), as discussed in Supplementary Note 5. This shows that the thickness of 4LG on HDM is ~2.1 nm (Supplementary Fig. 3), well below that of the current commercial COC. Graphene is transferred without such an energetic process as that used for commercial COC deposition. Thus, it does not adversely affect the magnetic performance. Even considering that COCs slightly affect the HDM magnetic properties, the influence of graphene will be much less than commercial COCs deposited by energetic processes, where energetic atoms/ions (neutrals also) hit and participate in deposition onto the HDM. Reference ^64^ reported that 1LG grown by CVD on Co (and Ni) does not affect the magnetic properties of these materials. In order to confirm this in our case, we perform polar magneto-optical Kerr effect measurements on BM, BM covered with ~2–3 nm COC, and BM covered with 1–4LG. 1–4LG coating results in a small change in the hysteresis loop. The coercivity changes from 510 mT (BM) to ~545 mT (1–4LG and COC). The remanent magnetization is unchanged at ~97–98% of the saturation magnetization when compared to BM. The coercivity and the remanent magnetization of 1–4LG-coated BM are similar to BM+COC, indicating that graphene coating does not have any detrimental effect on the media, see Supplementary Note 6 for details.

### Laser irradiation stability

HAMR is not possible without laser heating. References ^22,25,65^ reported that FePt does not degrade upon laser heating, with lubricant and COC being the main concerns when it comes to laser irradiation. Consistent with refs. ^25,55^, here we use an IR laser to examine the 1–4LG irradiation stability. To test whether 1LG can withstand HAMR conditions we consider 1LG transferred on L1_0_-FePt-based HDM^25,27,55^ and on Si/SiO_2_. Reference ^55^ achieved *T*_c_ at ~1.3 mW/μm^2^ by optimizing aperture optics. Reference ^25^ suggested using a laser power density ~10^7^  W/cm^2^ and ~0.35 × 10^7^  W/cm^2^ for FePt-based HDM. We perform Raman measurements at 785 nm, the available Raman wavelength closest to that used in HAMR (~830 nm^25,55^), with a spot size ~1.24 μm, as determined by the razor blade technique, see Supplementary Note 7 for details. We vary the power density from ~1.3mW/μm^2^ (0.013 × 10^7^ W/cm^2^) to ~31.5 mW/μm^2^ (0.315 × 10^7^ W/cm^2^) in order to examine the laser irradiation-driven evolution of the SLG Raman spectrum under HAMR power densities.

We first consider Si/SiO_2_/1LG. We record spectra at different power densities for ~4 min to achieve a good signal-to-noise ratio. Figure 2c shows that 1LG on Si/SiO_2_ has no D peak even for the highest power density. Pos(G) downshifts from ~1588 to 1583 cm^−1^, indicating an increase in *T* ~ 312.5 K with respect to room temperature, by taking ~−0.016 cm^−1^/K as a shift of Pos(G) with *T*^66^. Figure 2d plots the data for 1LG on FePt HDM, with *I*(G) normalized to that at 1.3  mW/μm^2^. In this case, the FePt substrate is rotated at 4100  rpm on a circular track with a diameter ~4 mm to simulate rotating HDD conditions, with a 20 min acquisition time, much larger than the total laser irradiation time expected for a 5-years life of HAMR-based HDDs^29^. No D peak is seen for all power densities, thus confirming the stability of 1LG. The Raman data show that no visible changes occur during the irradiation test measurements.

### Friction and wear

The HDM comprises magnetic/metallic layers, including Co-alloys-based magnetic storage layers^14,20^, with a high COF ~0.6–0.8^11,15^ and wear^11,15^. Therefore, they can experience mechanical damage whenever intermittent contact occurs with the head^11,15^, and are susceptible to corrosion^8,11,14^ and oxidation^11,15^, leading to HDD degradation or damage. The current ~ 2.7 nm commercial COCs have a high COF ~ 0.3–0.5^11,15^ and wear in a ball-on-disk tribological environment^11,15^, which can result in damage, hence durability concerns, see Supplementary Note 1 for more details.

Reference ^11^ used FCVA to deposit ~1.5–2 nm carbon films for protection of Co-alloy-based HDM and reported low COF ~0.25, wear, and corrosion. Here, we use 1–4LG grown by CVD for Co-alloy-based and HAMR HDDs.

Since in HDDs the HDM spins during operation, COF and wear need to be examined in a setup mimicking HDD operation. AFM and other tip-based tools are used to measure COF and wear^39^. In contrast to setups with rotating geometry, tip-based tools measure the COF based on the movement of the tip in the lateral direction^39^. Ball-on-disk measurements (rotation-based geometry) have a similar assembly as HDDs, with samples rotating while the counterface is in contact with the surface^11,35^, thus measuring  COF. We perform ball-on-disk tests using a nano-tribometer (CSM Instruments) in a cleanroom, to have a controlled environment with *T* = 23 ± 1 °C and a relative humidity ~55 ± 5%. A sapphire ball (Al_2_O_3_) of diameter ~2.0 ± 0.1 mm and surface roughness ~5.0 ± 0.1 nm is used as the counterface because the hard disk head is made of an Al_2_O_3_-based composite^6^. During the test, a normal load ~20 mN and a rotational speed ~100 rpm are used, corresponding to a linear speed ~1.05 cm/s for 10,000 cycles.

Since in HDDs the contact occurs occasionally^6^, the 10,000 cycles in our setup are much higher than the HDD operational lifetime. After each test, the wear track and ball images are captured using an optical microscope. To check repeatability, tests are performed 2–7 times. When two surfaces are in contact, and at least one of the surfaces starts to slide with respect to the other, friction and wear occur^67^. As a result, a wear track is formed.

**Fig. 3:**
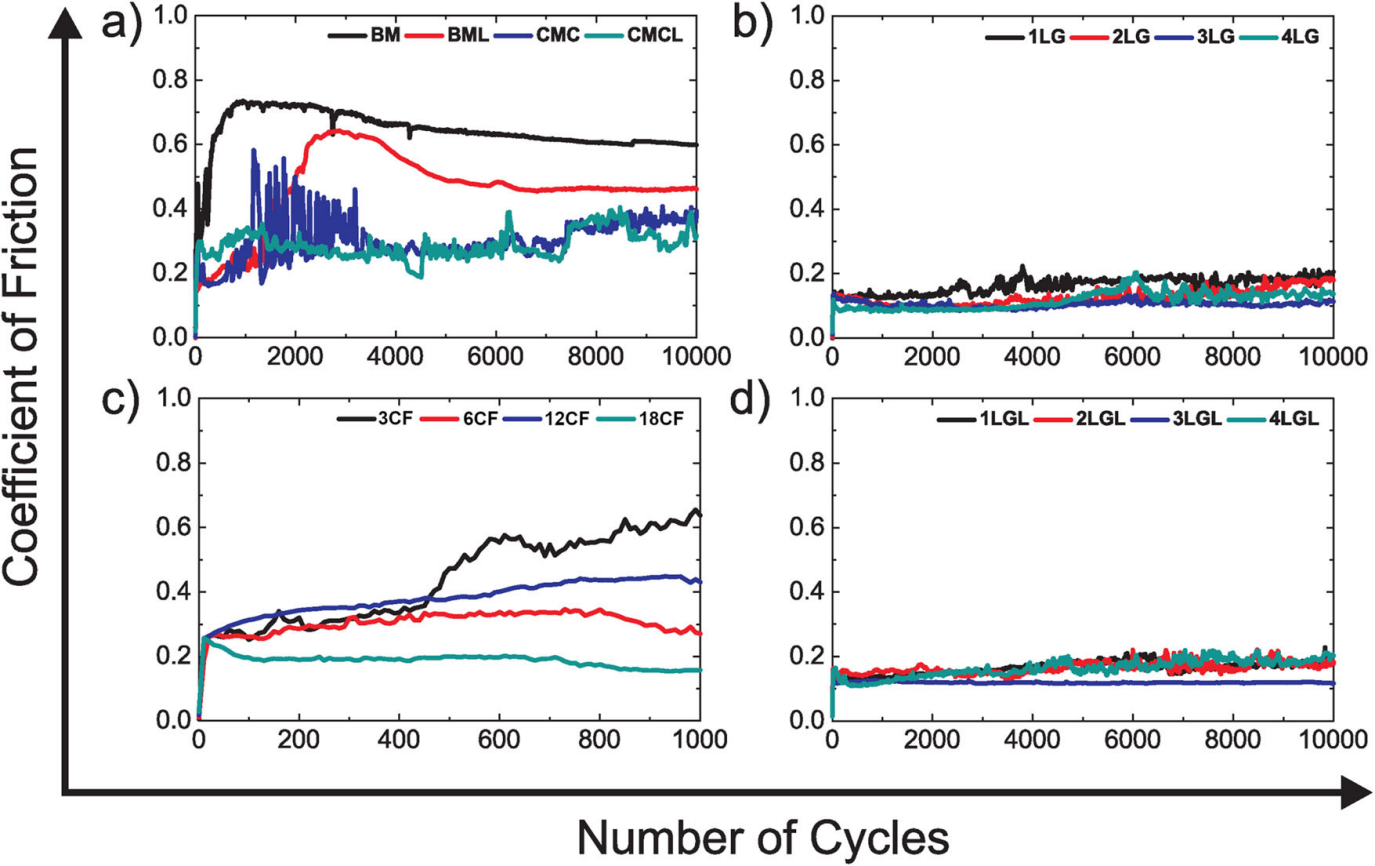
Friction measurements. Representative friction curves for (**a**) BM, commercial medium with commercial ~2.7 nm COC (CMC), commercial medium with commercial ~2.7 nm COC and ~1.0 nm PFPE lube (CMCL), BM coated with ~1.2 ± 0.2 nm PFPE lube (BML). **b** HDM coated with 1–4LG. **c** HDM with FCVA-ta-C of different thicknesses with 0.3 (3CF), 0.6 (6CF), 1.2 (12CF), and 1.8 nm (18CF) FCVA-deposited ta-C. **d** HDM coated with 1–4LG and ~1.2 ± 0.2 nm PFPE lubricant (L).

**Fig. 4:**
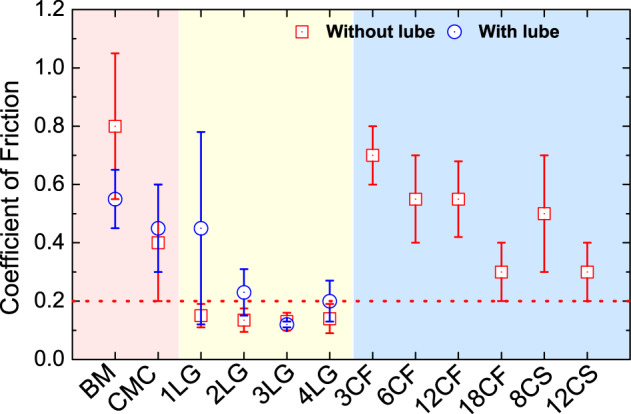
Comparison of measured coefficients of friction. Average COF from measurements in Fig. 3, with and without lubricant, for 1–4LG and all COC reference samples. The red shaded region highlights the bare media and media with a commercial overcoat, the yellow region highlights media with graphene overcoat, and the blue region highlights media with FCVA-ta-C. The horizontal red dashed line is a reference at COF = 0.2. The error bars indicate the spread around the average COF.

**Fig. 5:**
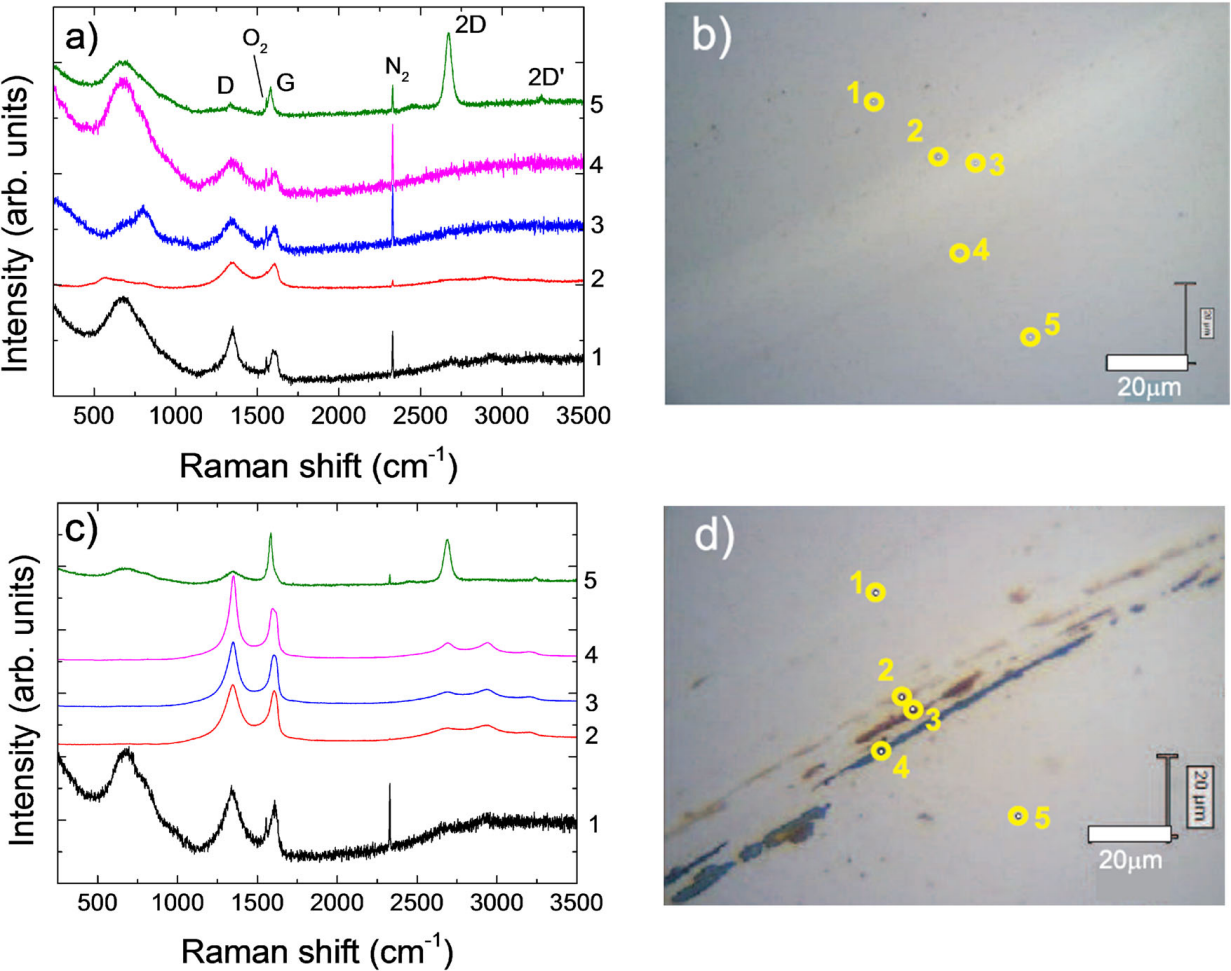
Raman measurements across wear tracks. Raman spectra, normalized to *I*(G), recorded across wear tracks of (**a**) 1LG and (**c**) 2LG and corresponding optical pictures in (**b**) and (**d**). In (**b**), (**d**) The Raman spectra are recorded across the wear tracks at the positions indicated by the yellow circles to compare graphene before and after friction and wear tests. The colors of the traces are due to a mixture of graphene, HDM components, and polymer residues coming from graphene transfer.

**Fig. 6:**
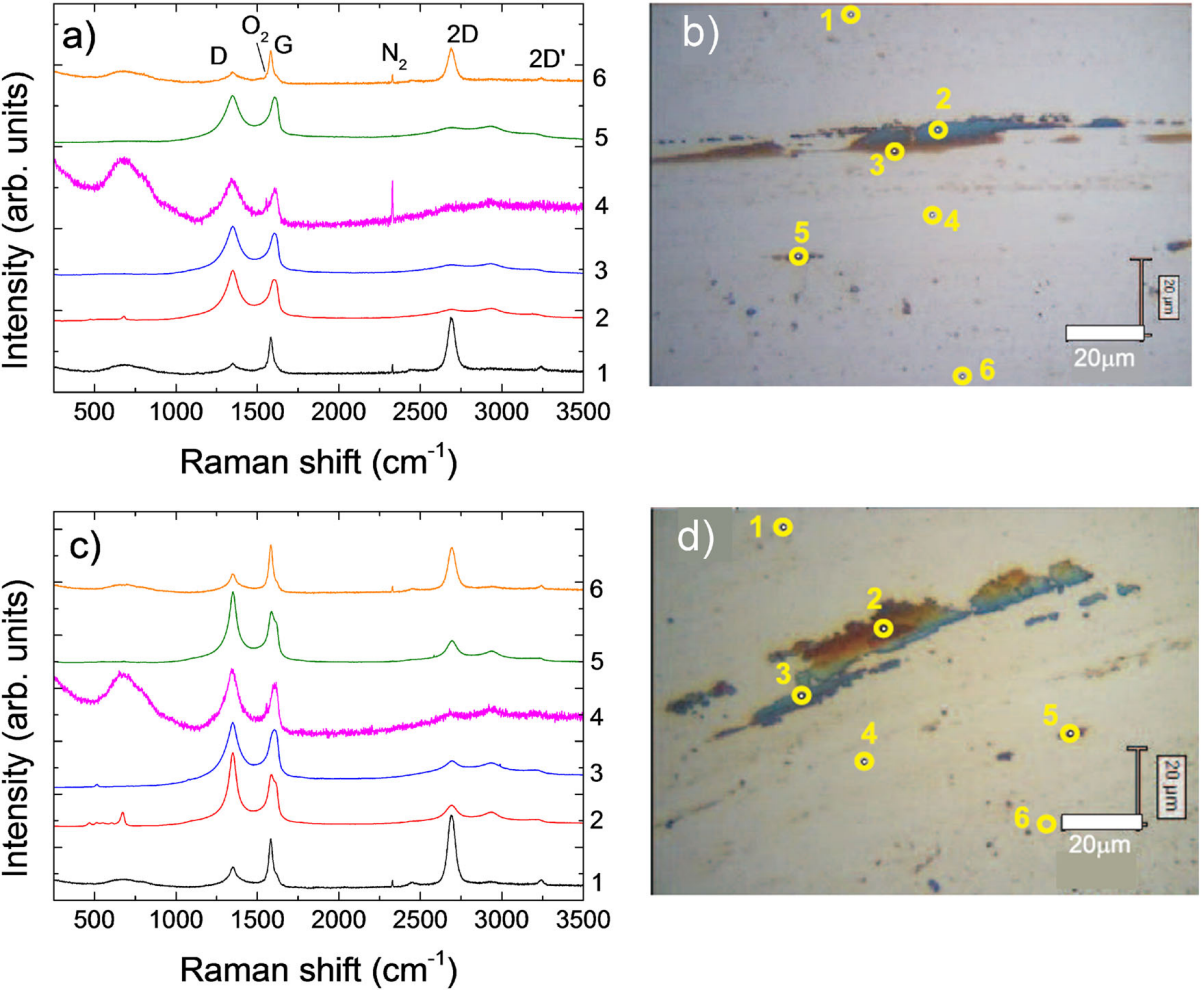
Raman measurements across wear tracks. Raman spectra, normalized to *I*(G), recorded across wear tracks of (**a**) 3LG and (**c**) 4LG and corresponding optical pictures in (**b**) and (**d**). In (**b**), (**d**) The Raman spectra are recorded across the wear tracks at the positions indicated by the yellow circles to compare graphene before and after friction and wear tests. The colors of the traces are due to a mixture of graphene, HDM components, and polymer residues coming from graphene transfer.

Figure 3 plots representative friction curves for BM, CMC, CMCL, and BML (Fig. 3a) coated with 1–4LG (Fig. 3b), and BM coated by FCVA (Fig. 3c). The average COFs are in Fig. 4, including  HDM coated by DC-sputtering. BM has the highest COF ~ 0.8, with substantial wear, as confirmed by optical images of balls and wear tracks in Figs. 5 and 6. The COF of ~2.7 nm CMC reaches ~0.4 at 10,000 cycles, but with strong fluctuations between ~0.2 and 0.6 at 1500 up to ~3500 cycles (Fig. 3a), and negligibly improves wear with respect to BM (Fig. 3a). The transfer of 1–4LG reduces COF < 0.2 for all samples, and gives non-fluctuating, smooth friction curves. The COF for 1–4LG-coated samples (without lube) is ~4 times lower than BM and ~2 times lower than CMC, despite a reduction of thicknesses ~7 times (for 1LG) to 2 times (for 4LG), using the theoretical 1LG thickness, with respect to CMC. Figures 5b, d and 6b, d reveal that the wear track width of 1–4LG-coated samples is ~2–4 times lower and debris transferred to the ball are smaller than BM and CMC, indicating higher wear resistance. All FCVA COCs with thicknesses from ~0.3 to ~1.8 nm show ~2–5 times higher COF than 1–4LG-coated samples, with and without lube, apart from 1LG without lube. Pulsed DC sputtered COCs have ~2–3 times higher COF than 1–4LG-coated samples.

To further analyze the wear tracks, Auger Electron Spectroscopy (AES) imaging is performed using a JEOL JAMP Auger Microprobe, see Supplementary Note 4 and Supplementary Fig. 1 for details. Before AES, scanning electron microscope images are taken to select the AES locations. The AES images inside and outside the wear tracks show the carbon-containing sites, and indicate that the amount of carbon on the wear track increases with increasing number of graphene layers, N. The Co and Cr intensities inside the wear tracks are higher for 1LG, and decrease with increasing N, due to the increase in C and the <1–3 nm sampling depth of AES^68^. The O signal in the wear track appears due to ambient oxygen as the samples are exposed to air before AES, with some contribution from the HDM oxide.

**Fig. 7:**
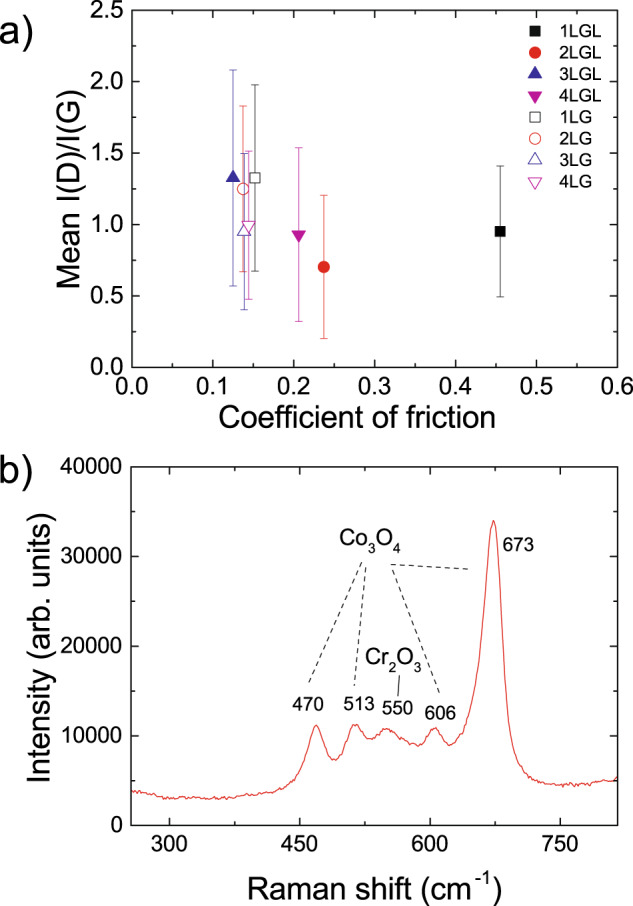
Raman analysis of defect-induced peaks and hard disk media. **a** Mean I(D)/I(G) of 1–4LG-coated samples, with and without lube, after friction tests, as a function of COF. The error bars come from averaging over the intensity ratios of the peaks in the Raman spectra recorded across the wear tracks. **b** Raman spectrum on a wear track. The region ~400–750 cm^−1^ shows peaks of the underlying substrate.

After ball-on-disk tests, the wear tracks are analyzed by recording Raman spectra across the wear track at different positions. Reference ^58^ introduced a three-stage model of amorphization. Stage 1: graphene to nanocrystalline graphene. Stage 2: nanocrystalline graphene to low *sp*^3^ amorphous carbon. Stage 3: low *sp*^3^ to high *sp*^3^ amorphous carbon. For all spectra in Figs. 5 and 6, as the D peak increases, D′ and D+D′ appear, whereas the *I*(2D) weakens when approaching the wear track, indicating an increase in disorder according to stage 1. The broad peak between 500 and 1000 cm^−1^ is due to the glass substrate^69^. At the center of the wear track, all second-order Raman features (i.e., 2D, 2D′, and D+D′) merge, while *I*(D) decreases and D,G become broader, indicating an increase of disorder^58^. 2–4LG is less damaged than 1LG, as for the spectra in Fig. 5a, c and *I*(D)/*I*(G) in Fig. 7a, from at least 5 positions as a function of COF, without and with lubricant (L). Overall, there seems to be no significant trend of *I*(D)/*I*(G) with COF. However, a difference is seen without lubricant: I(D)/*I*(G) is ~0.95 and ~1 for 3–4LG, while for 1–2LG it is ~1.33 and ~1.25. Thus, there are less defects in 3–4LG compared to 1–2LG. The peaks <1000 cm^−1^ in Fig. 7b are due to the HDM: those ~470 cm^−1^ (E_*g*_), 513 cm^−1^(F_2*g*_), 606 cm^−1^(F_2*g*_), 673 cm^−1^ (A_1*g*_) are from Co_3_O_4_^70,71^, while the A_*g*_ mode ~550 cm^−1^ is from Cr_2_O_3_^72^.

### Effect of lubricant

Current commercial HDDs use a layer of perfluoropolyether (PFPE) lubricant (lube)^11^ on top of COCs to further reduce friction and wear, and minimize surface energy. Given the lubricating and corrosion protection properties of 1LG itself, lube may not be needed for 1LG. However, exploring the compatibility of 1LG with lube is useful from the fundamental viewpoint.

Friction measurements on lube-coated samples (Fig. 3) show that in certain cases the COF is higher with L, than without. Taking the error bars into account, a significant difference in COF is only observed for 1LG and 1LGL. 1LG has wetting transparency^73^, i.e., it is thin enough that its introduction does not affect the wettability of an underlying substrate. Without overcoat, the L containing HDM, as in BML, shows very high COF ~ 0.55 and wear, due to the metal (medium) L-induced catalysis^74^. 1LG is not enough to avoid the interaction of L with medium, hence higher COF ~ 0.45 and wear is observed in 1LGL. Without L, 1LG shows very low COF ~ 0.15 and wear. A minor difference in case of 2LG versus 2LGL can be seen. However, for 3LG versus 3LGL and 4LG versus 4LGL the difference in average COF is marginal. L on 1LG results in higher and inconsistent COF and wear, as some measurements show lower COF ~ 0.15, some higher ~0.78, with COF increasing after few thousands cycles. The COF of 1LG, on an average, is ~0.45, i.e., ~3 times its non-lubricated counterpart. Thus, COFs of 2–4LG are more or less similar to those of the non-lubricated counterparts, suggesting that 2–4LG without L are lubricious enough, and that L does not improve lubricity. The D and G peaks in the Raman measurements in Figs. 5 and 6 on 1–4LG confirm the presence of carbon on the wear tracks, and transfer of debris to the balls, similar to the non-lubricated counterparts.

### Corrosion

Co-alloys have a great propensity to corrode, mainly due to Co oxidation^75^. This results in the loss of magnetic properties^75^, hence this is one of the major concerns for the long-term functionality and durability of HDDs^14,51,75^. To examine the corrosion protection efficiency of 1–4LG and compare their performance with state-of-the-art COCs, the corrosion of different uncoated- and coated-HDM, exposed to an electrolyte solution ~0.1 M NaCl similar to that used in refs. ^75^ on a ~0.24 cm^2^ area, is investigated using an electrochemical corrosion method^51,75^. The measurements are performed with a 3-electrode setup with a Pt wire as counter electrode, Ag/AgCl as reference electrode and HDM as working electrode, to which the potential is applied^51^. Each test consists of anodic and cathodic sweeps, where the potential is varied and the corresponding current measured^51^. Every sweep is conducted at different locations, with at least 3 sets of 6 sweeps on each sample. The so-called Tafel’s analysis^75^ is done by plotting anodic and cathodic curves on a semi-logarithmic scale of potential versus log current. The linear part of the logarithmic anodic and cathodic currents are extrapolated^75^, and the intercept of these lines gives the corrosion current^75^, and the corrosion current density *J*_corr_ when divided by the contact area.

The corrosion protection efficiency (CPE) defines how efficient the COC is in protecting against HDM corrosion. This is defined as^51^:

1 $${\mathrm{CPE}}\,( \% )=\frac{{J}_{\mathrm{corr}}^{0}-{J}_{\mathrm{corr}}}{{J}_{\mathrm{corr}}^{0}}\times 100,$$

where ${J}_{\,\text{corr}\,}^{0}$ is the BM corrosion current density and *J*_corr_ that of coated HDM.

When a metal is exposed to a corrosive solution, it releases ions that leave behind electrons, which can be observed in an anodic reaction as^75^:

2 $${\mathrm{M}}\to {\mathrm{M}}^{n+}+n{e}^{-},$$

where M represents the metal and *n* the number of electrons released by it. For Co-alloy-based systems, the metal dissolution, which decreases the anode conductivity^75^, can be written as^75^:

3 $${\mathrm{Co}}\to {\mathrm{Co}}^{2+}+2{e}^{-},$$

As the corrosion reaction involves the transfer of electrons and ions between metal and solution^75^, the corrosion rate varies with corrosion current^75^, hence *J*_corr_ varies inversely with corrosion resistance, i.e., the COC ability to reduce the HDM corrosion^51,75^.

**Fig. 8:**
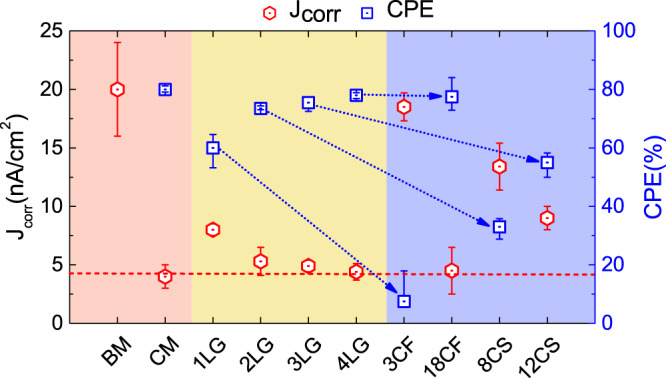
Corrosion measurements. Corrosion current density *J*_corr_ and corrosion protection efficiency (CPE) for different samples. The corrosion resistance varies inversely with *J*_corr_^51^. 2–4LG have similar CPE to ~2.7 nm CMC and ~1.8 nm COC in sample 18CF. The dotted line is a guide to the eye for *J*_corr_ of different samples. The arrow compares various 1–4LG-COCs pairs, such as 1LG → 3CF, 2LG → 8CS, 3LG → 12CS, and 4LG → 18CF, as the 1–4LG-based sample in each pair is always slightly thinner than the amorphous COC. The red shaded region highlights the bare media and media with a commercial overcoat, the yellow region highlights media with graphene overcoats and the blue region highlights media with FCVA-deposited ta-C. The horizontal red dashed line is a reference at *J*_corr_ = 4 nA/cm^2^. The error bars stem from taking the average of multiple repeated measurements.

The BM shows the highest *J*_corr_, indicating the greatest propensity to corrode (Fig. 8). Introduction of 1–4LG reduces HDM corrosion. *J*_corr_ decreases with *N*, from ~5.3 nA/cm^2^ for 2LG to ~4.4 nA/cm^2^ for 4LG, Fig. 8, and CPE increases. The COC defects and pinholes are the active corrosion sites^75^, and their electrical conductivity could further add galvanic corrosion^75^. The COC should be defect- and pinhole-free, smooth, and with excellent barrier properties to minimize HDM oxidation and corrosion. Reference ^43^ showed that 1LG can be used as a corrosion barrier for metallic surfaces, and suggested that this should be uniform and defect-free to achieve corrosion protection. In our case, 1LG reduces *J*_corr_ by ~2.5 times with respect to BM, but it is twice that of BM with ~2.7 nm COC. For 2LG, *J*_corr_ is ~3.8 times smaller than BM, and marginally higher than COC. This is remarkable as the 2LG thickness ~0.7 nm, is ~4 times lower than ~2.7-nm-thick COC. Beyond 2LG, *J*_corr_ and CPE remain similar, adding marginal anti-corrosion improvement (Fig. 8). The higher corrosion protection in 2–4LG with respect to 1LG is mainly due to the fact that the increase in N improves the HDM coverage, leading to the reduction of active corrosion sites. 2–4LG display corrosion protection similar to COC, attributed to better barrier properties. Figure 8 indicates that, for a similar thickness, graphene-based overcoats have lower *J*_corr_ than amorphous COCs, indicating greater protection. Figure 8 shows that the corrosion resistance performance of 1LG is ~2.5 times better than BM, but ~2 times higher than COCs. By adding graphene layers the corrosion resistance is improved. The difference in corrosion current density between COC and 2–4LG is 30–10%. Thus, the corrosion resistance of 2–4LG approaches that of COCs. Taking into account that the theoretical thickness of 1LG is ~8–9 times lower than state of the art COCs, and that a COC having the same thickness as 1LG would provide no protection and be full of holes^19^, 1LG comfortably beats any COC of the same thickness (i.e., same storage density).

## Discussion

Friction is defined as resistance to sliding^36,76^. At the macroscopic level, Amonton’s law^76^ states that the frictional force between two bodies varies proportionally to the normal force. Hence:

4 $${\mathrm{COF}}=\frac{F}{W},$$

where *F* [mN] is the frictional force and *W* [mN] is the normal force. This does not take into account the area of contact at the microscopic level^76^. Reference ^76^ suggested that the contact between two bodies contains several smaller contacts, called asperities, with the sum of the areas of these asperities being lower than the apparent macroscopic area^76^. Thus, from ref. ^76^, *F* in micro-tribology can be expressed as:

5 $$F=\tau \sum {A}_{\mathrm{asp}},$$

where *τ* is the shear strength, i.e., the stress required to shear the contacting interfaces and enable sliding, expressed as shear force/area and ∑*A*_asp_ is the sum of the areas of the asperities, also called real contact area^76^. Hence, friction also depends on ∑*A*_asp_. Reference ^76^ showed that the stresses over regions of contact reach the elastic limit of the material and cause plastic deformation. Thus, the average pressure (*p*) in a contact region is governed by the normal force, and is given by *p* = *W*/*A*^77^. The zone of deformation increases with increase in load, and *p* on the region of contact tends to a stable value, which eventually causes the deformation to become entirely plastic. The process continues until the area of contact becomes sufficient to support the load *W*. Consequently, the normal force can be expressed as:

6 $$W=p\sum {A}_{\mathrm{asp}},$$

Thus, Eqs. (4)–(6), give:

7 $${\mathrm{COF}}=\frac{F}{W}=\frac{\tau }{p}$$

A co-alloy is mechanically softer^78^ than Al_2_O_3_^79^. When a metallic co-alloy surface slides against Al_2_O_3_, it causes the HDM surface to undergo plastic deformation^76^, generating wear and high COF ~0.8, Figs. 3 and 4, consistent with the friction results for metallic surfaces in ref. ^76^. This could be due to the formation of an adhesive contact at the HDM-ball interface, due to the high surface energy of Co-alloy-based HDM ~ 42.8 mN/m^80^, and the presence of contaminants at the interface that may enhance the interaction between the two bodies^81^. When 1LG is placed on HDM, the COF decreases ~4 times and negligibly changes with *N*.

At the nanoscale, the friction of 1LG on Si/SiO_2_ against Si tips, measured at a low load ~1 nN and scan speed ~1–10 μm/s, was explained based on out-of-plane deformation in front of a scanning probe tip, the so-called puckering effect^39,82^. This enhances the contact area, hence friction. The out-of-plane deformation is suppressed with increasing N^82^, resulting in reduced COF. This is not applicable to ball-on-disk tribological tests of 1–4LG, due to the significantly larger dimension (ball radius ~ 2.0 ± 0.1 mm) of the counterface, much higher load ~20 mN and higher speed ~100 rpm or 1.05 cm/s, which do not precisely differentiate the frictional characteristics of 1–4LG. Thus, all coated media show similar friction (Fig. 3b). Since the layers are coupled by van-der-Waals forces, they shear easily, as their interfacial shear strength is low, with a shear force per unit area *α* ~ 12.8 ⋅ 10^18^ N/m^3^ ^83^ and a resulting shear modulus *C*_44_ ~ 4.3 GPa^83^. The ease of shearing at sliding contacts results in smoother frictional curves (and marginally lower COF) in 2–4LG with respect to 1LG. The improved wear resistance for 1–4LG-coated HDM is attributed to reduced friction, wear, and excellent mechanical properties of 1LG: breaking strength ~42 N/m^84^, Young’s Modulus ~ 1 TPa^84^, flexibility (1LG can be stretched up to ~20% without breaking^84^).

When compared to BM, the COF reduction of 1–4LG-coated HDM could be attributed to reduced adhesive interaction (lower surface energy)^36,85^, as well as incommensurability of the lattice planes sliding against each other at the tribological interface^35^, occurring when the hills of a surface with lattice spacing *a* do not match the valleys of another surface of lattice spacing *b*, such that *b*/*a* is an irrational number^35^. This is consistent with what suggested in ref. ^35^ for 3–4LG on Si/SiO_2_ sliding against an a-C:H-coated steel ball, and for 1–3LG on a steel sliding against a steel ball^35,37^ in macroscale ball-on-disk conditions, e.g., with a normal force in the *z* direction *N*_*z*_ ~ 0.5–3 N, and speeds ~0.6–25 cm/s.

The AES on the wear tracks and Raman measurements across the wear tracks, after ball-on-disk tribological tests, in Figs. 5 and 6 show that a C signal is still present in all 1–4LG samples, even though disorder-induced peaks appear. Reference ^86^ also found C on wear tracks after microscale friction and wear tests of graphene on SiC. The counterface also reveals transferred debris, consisting of disordered carbon and underlying substrate atoms. This implies that, when a 20 mN load is applied and the sample starts to rotate at ~2.1 cm/s, 1LG could be turned into patches, due to the large contact load. Reference ^86^ reported transformation of continuous layers of 1–2LG into 1–2LG patches during microscale tribology, despite using lower load ~0.1–1 mN and speeds ~30–50 μm/s compared to us. This led to a distribution of 1LG patches along the wear track, and transfer of 1LG-containing debris on the counterface. This also happens for 2–4LG-coated HDM. Raman measurements in Figs. 5 and 6 show disordered carbon on the wear tracks, with disorder lower than for 1LG, as revealed by *I*(D)/*I*(G) (Fig. 7). This implies that 1LG acts as lubricant when in contact with another 1LG, and is not completely removed during tribological tests. AES also reveals a progressive increase in C and C-containing sites on the wear tracks with increasing number of layers. The debris transferred to the ball also contain C. Therefore, the formation of disordered carbon debris on both surfaces facilitates smooth sliding, and contributes to maintaining the low COF. The marginally lower friction in 2–4LG as compared to 1LG can be linked to *I*(D)/*I*(G) (Fig. 7a), with lower disorder corresponding to lower friction, although this does not apply for 1–4LGL.

**Fig. 9:**
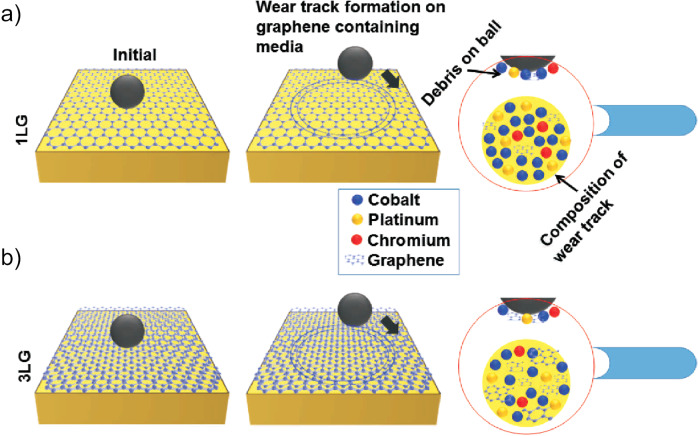
Friction model. Interaction of counterface ball (sapphire) with (**a**) 1LG and (**b**) 3LG. The zoomed-in images represent 1LG and 3LG, and underlying substrate atoms (Co, Pt, Cr, etc.) on counterface ball and wear track, where the amount of C debris increases with *N*. The debris generated on both interacting bodies maintain the lower COF of 1–4LG with respect to BM.

Figure 9 is the proposed mechanism for friction reduction for 1LG and 3LG-coated HDM, where C debris are on both ball and wear track. To validate these assumptions, we perform molecular dynamics (MD) simulations of a Co∣Pt–sapphire system with and without 1 and 4LG, as discussed in Supplementary Note 8.

**Fig. 10:**
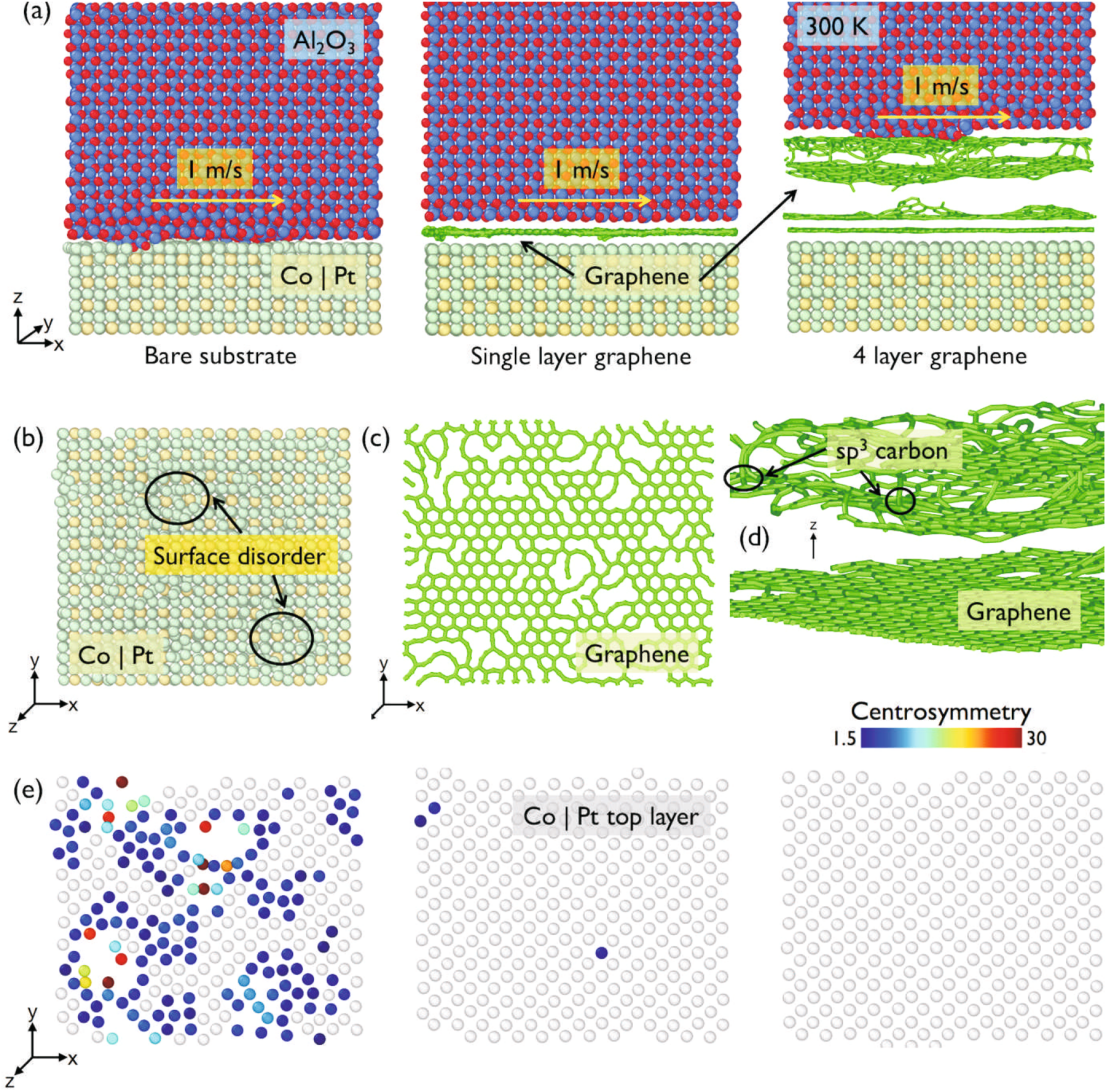
Simulations of friction process. Light green spheres indicate Co∣Pt, red and blue spheres indicate Al_2_O_3_. **a** Simulation setup for evaluating wear between Al_2_O_3_ and Co∣Pt. Simulations are performed for BM, 1, 4LG at 300 K, with materials sliding against each other at 1 m/s. **b** Interfacial surface atoms of bare Co∣Pt showing disorder. Two representative disorder regions are indicated by circles. **c** Structure of defected 1LG. **d** Structure of 4LG. The top two layers are attracted towards the Al_2_O_3_ block with the formation of *sp*^3^ cross-links. **e** Disorder of interfacial Co∣Pt surface using the centrosymmetry parameter.

The COF is estimated as the ratio of sliding and normal force, block averaged every 25 ps. Figure 10a–e compares the computed COF and surface disorder of Co∣Pt, 1LG, and 4LG. The surface disorder represents the friction-induced damage/wear of the HDM, and its value is derived considering 0% surface disorder before friction measurements. The amount of disorder in HDM is quantified using the centrosymmetry parameter, a measure of the local lattice disorder around an atom. This is 0 for a perfect lattice, whereas when a point defect exists, i.e., when the symmetry is broken, it assumes a larger positive value, see Supplementary Note 8 for details. The simulations indicate that BM develops substrate disorder up to ~14.5% within 350 ps (Fig. 10b, e).

High wear is also observed for BM in the experiments in Fig. 3a. An additional 250 ps where the forces on atoms are also collected are used to obtain the COF (Supplementary Fig. 5). The COF averaged over these 250 ps is ~0.82 ± 0.1. 1LG reduces the average simulated COF to ~0.18 ± 0.02. Figure 10c shows that 1LG maintains its structural integrity with fraction of disorder ~1% on average, as a consequence of the low COF between the blocks, indicating higher resistance to wear as compared to BM. 4LG further improves the tribological behavior. The COF drops slightly to ~0.14 ± 0.0.02 and substrate disorder is reduced to ~0.1%, much lower than in BM, suggesting that the BM surface remains mostly unaffected. Simulations suggest partial transfer of graphene patches to the sapphire ball. The Raman analysis of wear tracks in Figs. 5 and 6 shows tribo-induced disorder in 1–4LG transfer of C to the ball. Thus, the patches containing debris on both surfaces are responsible for the lower COF in 1–4LG-coated HDM, maintaining higher wear resistance than BM.

Reference ^87^ showed that potential corrugations on sliding surfaces can impact friction force and anisotropy of MLG. It reported that higher friction forces result from larger corrugations of the potential energy and depend on *N*. This is consistent with our experiments in Figs. 3 and 4, and simulations in Fig. 10.

In summary, we analyzed 1–4LG-coated media and found that they can overcome the tribological and corrosion issues of current Co-alloy-based HDM, with laser irradiation stability on FePt-based HDM for HAMR. The overall performance of graphene-coated media exceeds that of thicker commercial COC, as well as other amorphous carbons of comparable/higher thicknesses prepared by FCVA and sputtering. Given the tribological, corrosion, and thermal stability characteristics coupled with an AFM thickness ~4 times (for 1LG) to ~1.3 times (for 4LG) lower than state-of-the-art 2.7-nm COCs, we expect 1–4LG-based overcoats to meet the requirements for 4–10 Tb/in^2^ areal density HDDs, when employing suitable recording technologies, such as HAMR and HAMR+BPM. Graphene-coated media have better corrosion and wear resistance with a COF < 0.2. A single layer of graphene is enough to reduce corrosion ~2.5 times and can withstand HAMR conditions. Our results imply that >2LG-based coatings could be used as a tribological interface for various other materials/devices, such as micro- and nano-electromechanical systems.

## Methods

### Graphene growth and transfer

1LG is grown by CVD on a 35 μm Cu foil. The substrate is loaded into a hot wall tube furnace evacuated to ~1 mTorr. The Cu foil is annealed in hydrogen at 1000 °C for 30 min. This reduces the copper oxide surface and increases the Cu grain size. The growth process starts when 5 sccm CH_4_ is added to H_2_. After 30 min the substrate is cooled down for several hours in vacuum (~1 mTorr) to room temperature and unloaded.

We use a polymethyl methacrylate (PMMA)-based wet transfer^52,53^. First, ~500 nm PMMA is spin-coated on the sample. The PMMA/1LG/Cu stack is then placed in an aqueous solution of ammonium persulfate to etch Cu^52^. When Cu is fully etched, the graphene/PMMA-stack is placed into a de-ionized (DI) water bath to rinse any acid residuals and subsequently fished out using the HDM substrate. After drying for one day at room temperature, the sample is placed in acetone to remove PMMA, leaving 1LG on the HDM. By repeating the steps described above, several 1LGs are transferred to create an MLG stack. The same procedure is used to place 1LG and MLG onto FePt.

## Supplementary information

Supplementary Information

## Data Availability

All relevant data are available from the authors upon reasonable requests. Unique identifiers such as DOI and hyperlinks for any other data sets are also available from the authors upon reasonable request. Source data are provided with this paper.

## Source data

Source Data

## Code availability

All relevant custom software is available from the authors upon reasonable request
