# Publisher Correction: Graphene overcoats for ultra-high storage density magnetic media

**DOI:** 10.1038/s41467-021-23869-4

**Published:** 2021-06-02

**Authors:** N. Dwivedi, A. K. Ott, K. Sasikumar, C. Dou, R. J. Yeo, B. Narayanan, U. Sassi, D. De Fazio, G. Soavi, T. Dutta, O. Balci, S. Shinde, J. Zhang, A. K. Katiyar, P. S. Keatley, A. K. Srivastava, S. K. R. S. Sankaranarayanan, A. C. Ferrari, C. S. Bhatia

**Affiliations:** 1 CSIR-Advanced Materials and Processes Research Institute, Bhopal, India; 2 Department of Electrical and Computer Engineering, National University of Singapore, Singapore, Singapore; 3 Cambridge Graphene Centre, University of Cambridge, Cambridge, UK; 4 Department of Engineering, University of Exeter, Exeter, UK; 5 Center for Nanoscale Materials, Argonne National Laboratory, Argonne, IL USA; 6 Institute of Materials, Ecole Polytechnique Fédérale de Lausanne (EPFL), Lausanne, Switzerland; 7 Empa-Swiss Federal Laboratories for Material Science and Technology, Dübendorf, Switzerland; 8 Department of Physics and Astronomy, University of Exeter, Exeter, UK; 9 Department of Mechanical and Industrial Engineering, University of Illinois, Chicago, IL USA

**Keywords:** Materials science, Materials for devices, Nanoscale materials

Correction to: *Nature Communication*s 10.1038/s41467-021-22687-y, published online 17 May 2021.

In the original version of this Article, Figures 9 and 10 were erroneously swapped. This has now been corrected in the PDF and HTML versions of the Article.

