# Supplementary material for: Graphene overcoats for ultra-high storage density magnetic media
Source: Nat Commun. 2021 May 17;12:2854. doi: 10.1038/s41467-021-22687-y (PMC8129078; doi:10.1038/s41467-021-22687-y)
Supplement: Supplementary file 1 — Supplementary Information [file 41467_2021_22687_MOESM1_ESM.pdf]

Supplementary Information for Graphene Overcoats For Ultra-High Storage Density Magnetic Media

N. Dwivedi[1,2]*, A. K. Ott[3,4]*, K. Sasikumar[5], C. Dou[3], R. J. Yeo[2,6], B. Narayanan[5], U. Sassi[3], D. De Fazio[3], G. Soavi[3], T. Dutta[2,7], O. Balci[3], S. Shinde[3], J. Zhang[3], A. K. Katiyar[3], P. S. Keatley[8], A. K. Srivastava[1] S. K. R. S. Sankaranarayanan[5,9], A. C. Ferrari[3†], C. S. Bhatia[2]

[1] CSIR-Advanced Materials and Processes Research Institute, India 462026
[2] Department of Electrical and Computer Engineering, National University of Singapore, Singapore 117583
[3] Cambridge Graphene Centre, University of Cambridge, Cambridge CB3 0FA, UK
[4] Department of Engineering, University of Exeter, Exeter EX4 4QF, UK
[5] Center for Nanoscale Materials, Argonne National Laboratory, 9700 S Cass Avenue, Argonne IL USA
[6] Institute of Materials, Ecole Polytechnique Fédérale de Lausanne (EPFL), 1015 Lausanne, Switzerland
[7] Empa-Swiss Federal Laboratories for Material Science and Technology, Dübendorf, Switzerland-9014
[8] Department of Physics and Astronomy, University of Exeter, Exeter EX4 4QL, UK
[9] Department of Mechanical and Industrial Engineering, University of Illinois, Chicago, IL 60607
* These authors contributed equally
† email:acf26@eng.cam.ac.uk

Supplementary Figures

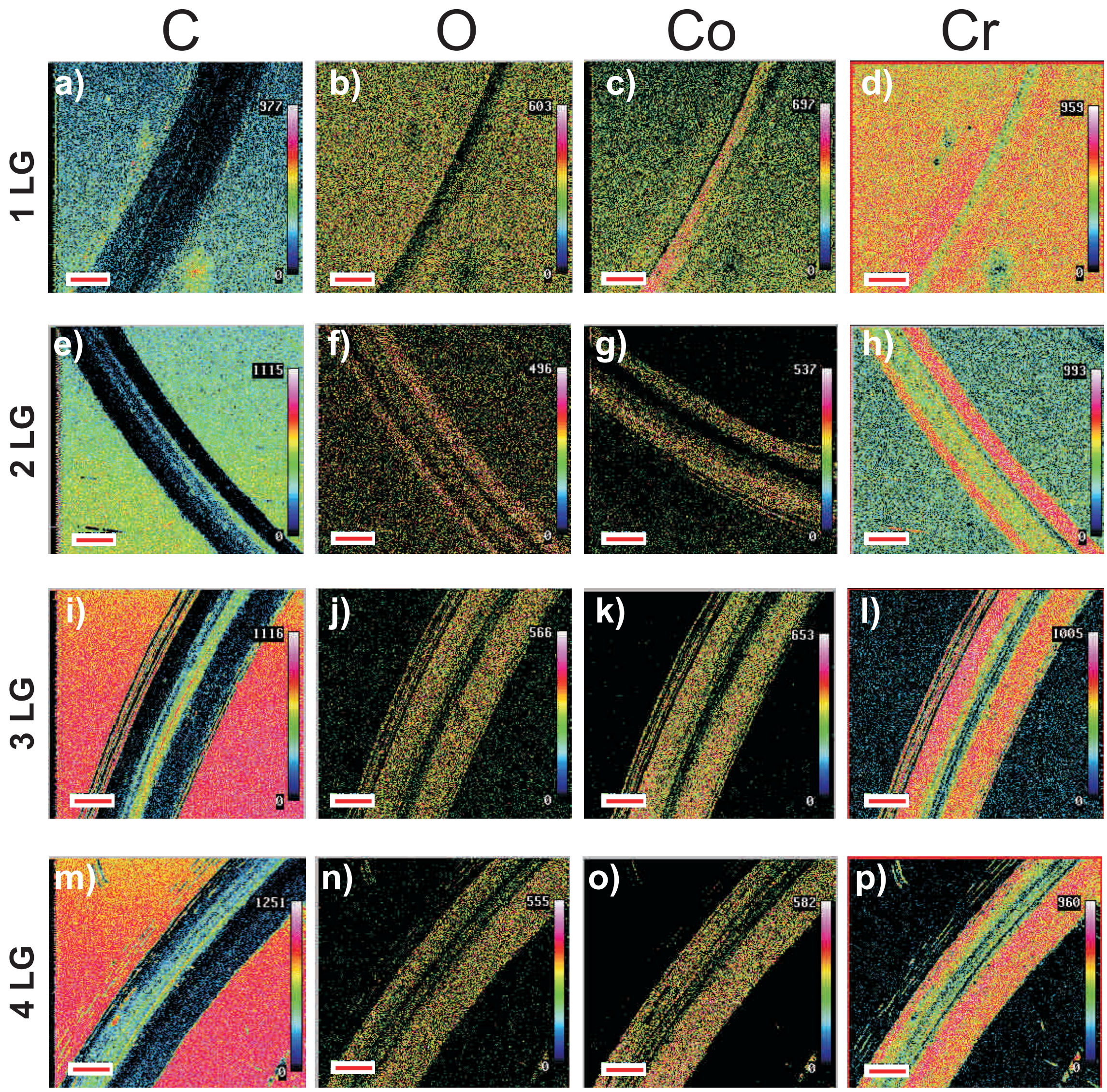

Supplementary Figure 1: AES images of C, O, Co, Cr for a-d) 1LG, e-h) 2LG, i-l) 3LG, m-p) 4LG. Brighter colors indicate a higher intensity. Outside the wear track, the C signal (top layer) increases and Co and Cr signals (underlying substrate) decrease with increasing N, due to limited AES sampling depth[1].

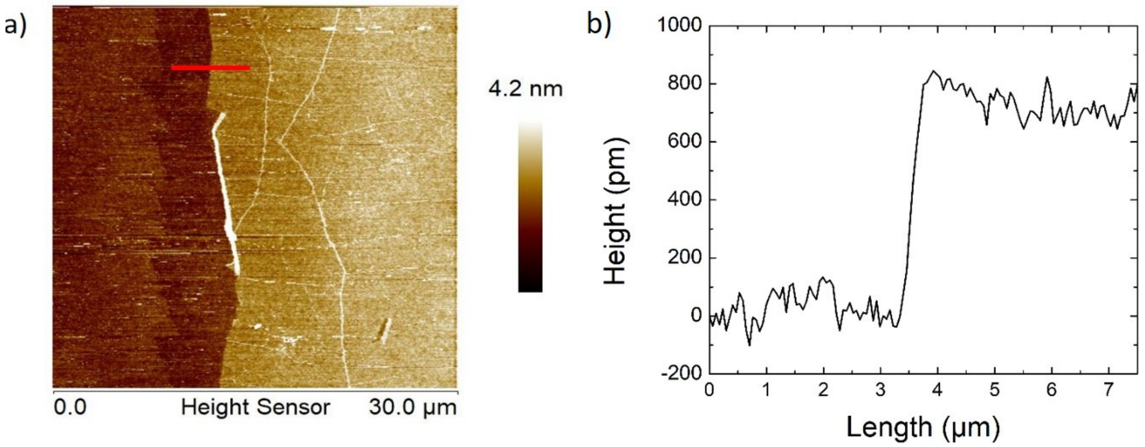

Supplementary Figure 2: a) AFM scan of CVD 1LG on Si/SiO_2. b) Height profile corresponding the red line in a).

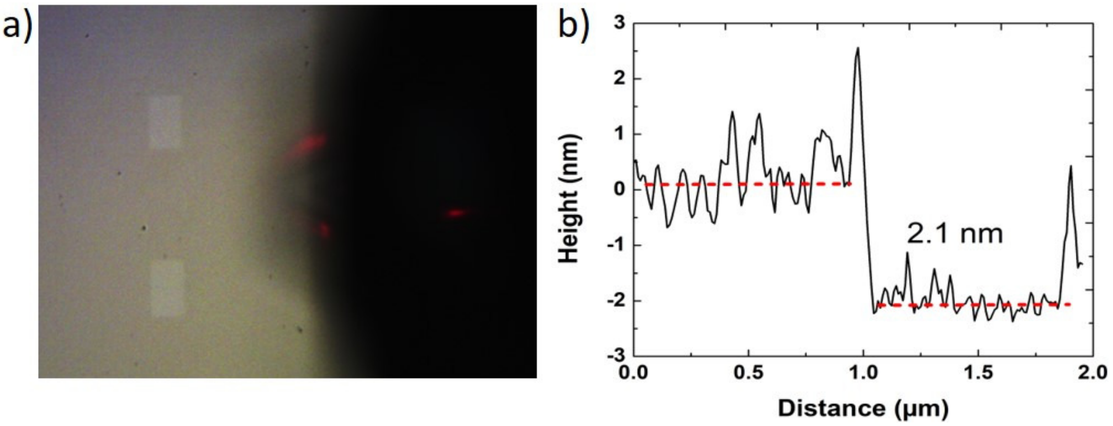

Supplementary Figure 3: a) Patterned graphene on HDM. b) AFM step height for 4LG transferred on HDM across the edge of the etched rectangle. The step height is~2.1nm

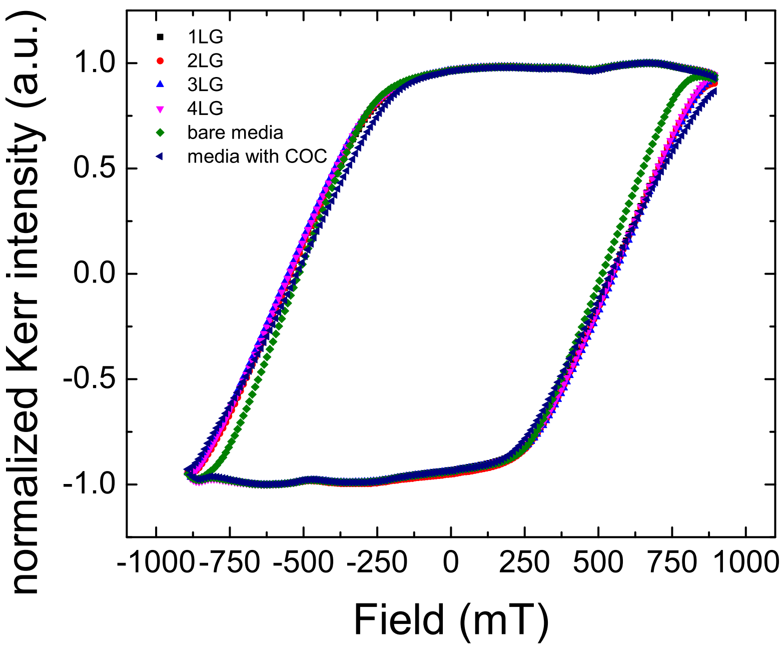

Supplementary Figure 4: Normalized Kerr intensity as function of applied field. The loops are recorded on BM, BM+COC, and BM+1-4LG.

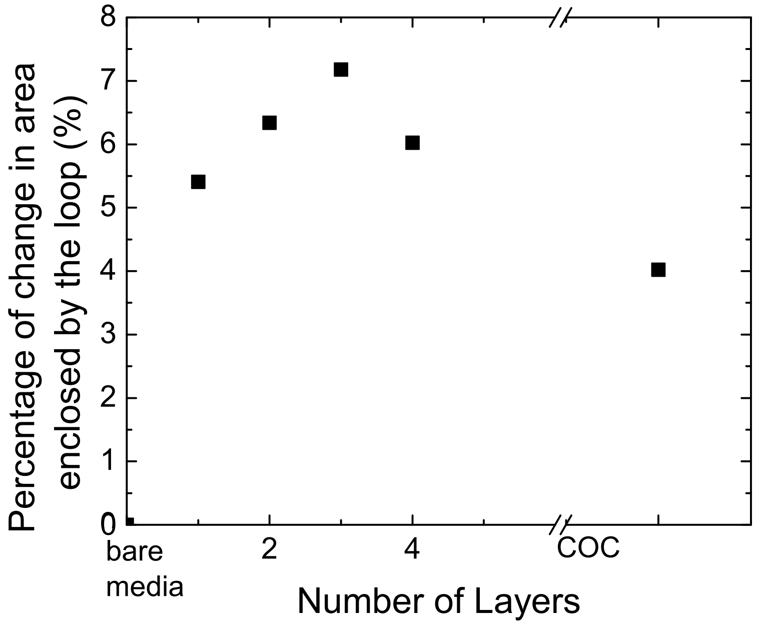

Supplementary Figure 5: Percentage of change in area enclosed by the hysteresis loops, with respect to that recorded on BM

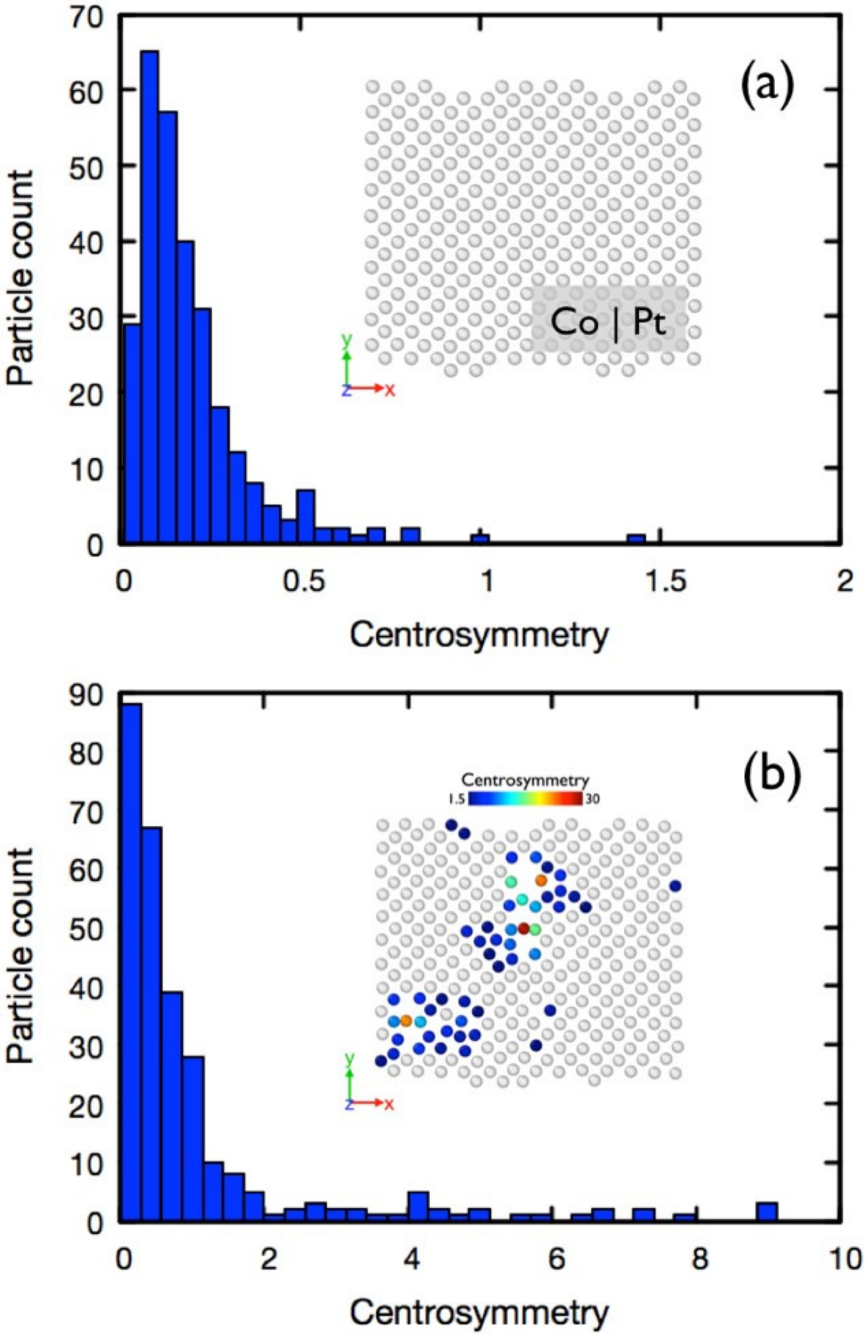

Supplementary Figure 6: Histogram of centrosymmetry parameter for Co|Pt surface layer in the interfacial region. (a) Defect-free structure with centrosymmetry parameter<1.5. (b) Defected surface. The atoms in the inset are colored by the centrosymmetry parameter for values≥1.5

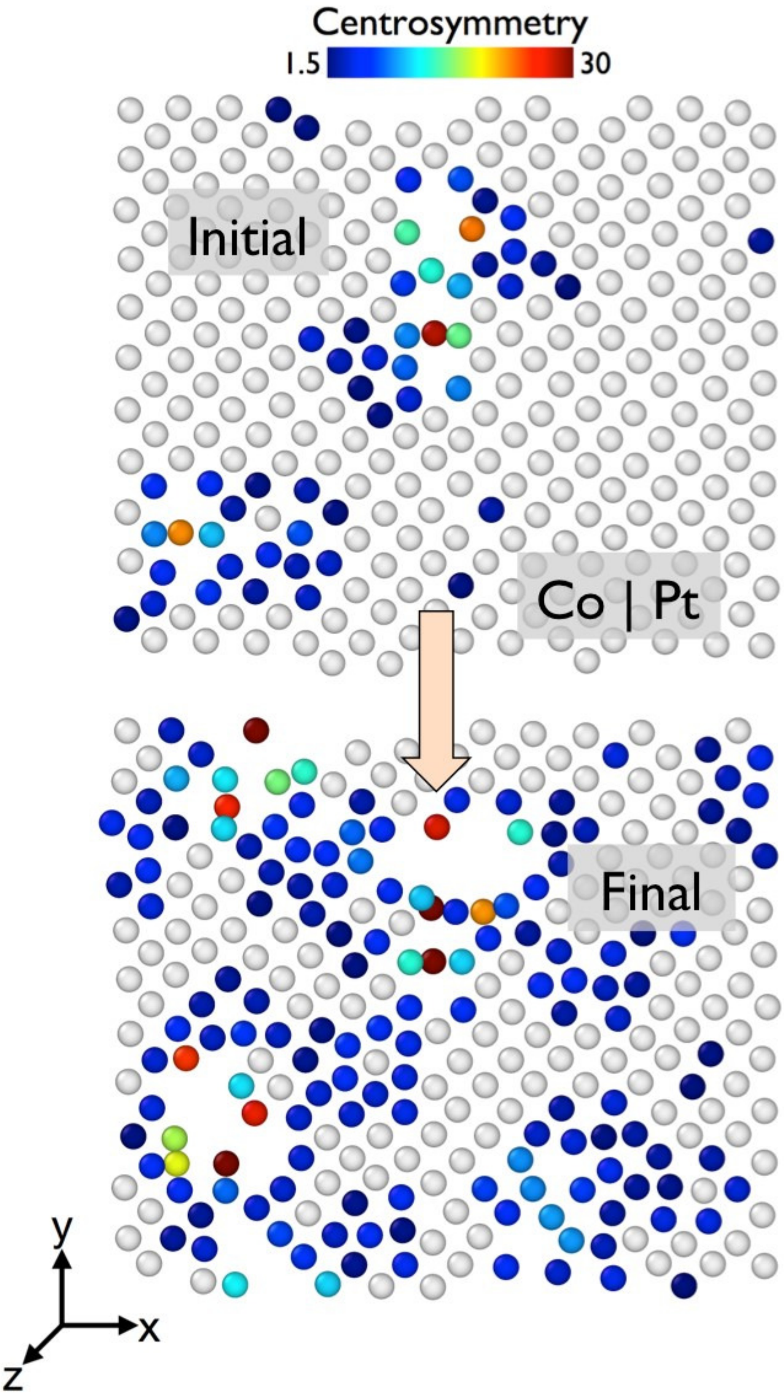

Supplementary Figure 7: Co|Pt top surface colored by centrosymmetry parameter. (a) Thermalized Co|Pt surface at interface with alumina before sliding. (b) Defected surface after 350ps of frictional sliding at 1m/s

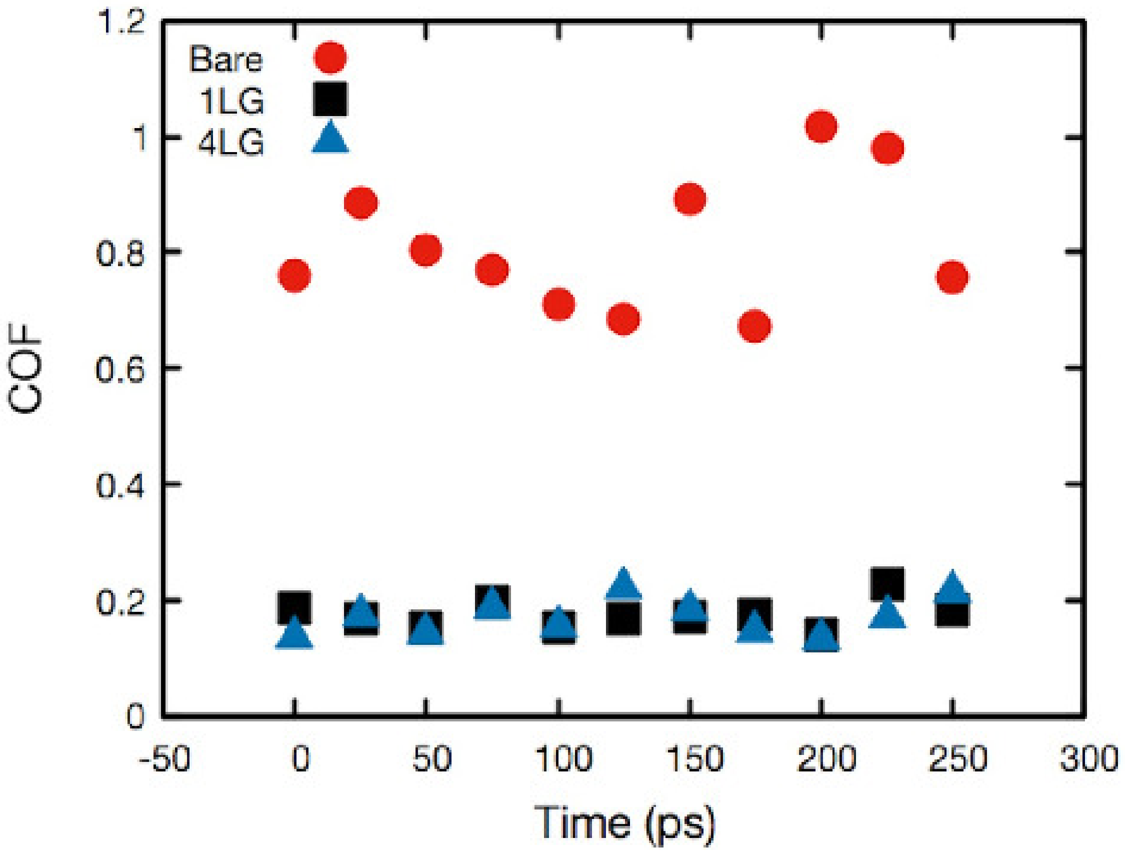

Supplementary Figure 8: Time evolution of friction coefficient for BM, 1LG, 4LG

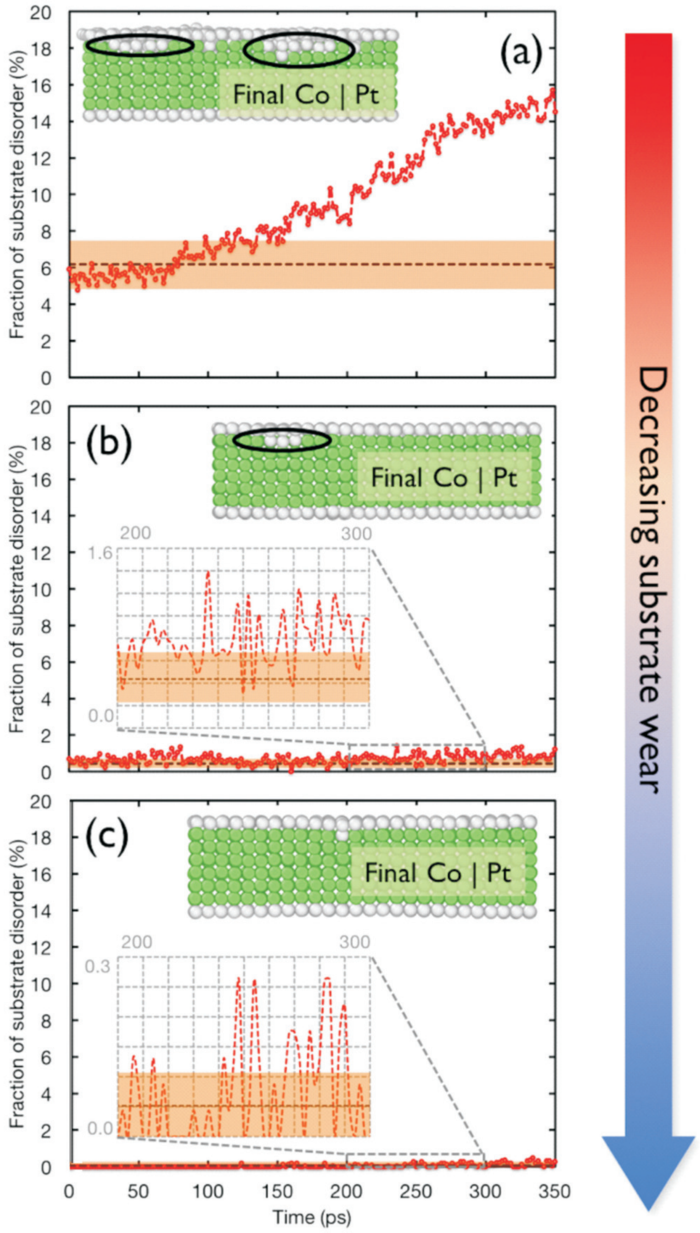

Supplementary Figure 9: Fraction of Co|Pt disorder for a) HDM, b) HDM+1LG, c) HDM+4LG. In (a-c) the horizontal dotted line represents the HDM disorder before friction at 300K by virtue of cross interactions between alumina, 1/4LG and substrate. The transparent orange box indicates the standard error in the fractional substrate disorder at 300K. The insets shows atomistic snapshots of Co|Pt at 350ps. The green atoms have a local FCC environment, while the white ones are surface and/or defects. The secondary insets in (b,c) report the variation of fractional disorder from 200-300ps

Supplementary Tables

Supplementary Table 1: LJ Potential parameters for the various pair-wise interactions

Interaction	ϵ (eV)	σ (Å)	Reference
C-C	0.003000	3.410	[2]
Pt-Pt	0.694000	2.471	[3]
Al-Al	0.408000	2.551	[4]
Co-Co	0.000304	2.872	[5]
O-O	0.007160	3.064	[6]

Supplementary Note 1: Hard disk technology background

HDDs will rule storage technologies at least for the next 5-10 years[7, 8, 9]. This is due to their low cost<0.1$/GB at 2016 prices[10] and large storage capacity>10TB with 3.5inch HDDs[10]. This is reflected in the 425 million HDDs shipped globally in 2016[11], decreasing to 350 million by 2020[11], due to the expected increase in storage capacity per drive.

The key components of HDDs are 1) hard disk medium (HDM)[12, 13]; 2) head[13]; 3) mechanics and servo[14]. In a HDD the information is written in/read from the HDM by the head[15]. To meet the rise of the data storage demand, HDD technology moved from inductive, prior to 1990[13], to magneto-resistive from 1990[13], and giant magneto resistive or tunneling magneto resistive from 1997[13] and 2004 onwards[13]. HDM technology moved from particulate media[12] in the first HDD generations in the late 1950s[12] to thin film media[16, 12] after their introduction in the 1980s into 5.35" drives[16, 12]. Servo and signal processing technologies also advanced.

To meet the rise of the data storage demand, HDD technology has moved from inductive, used prior to 1990[17], to magneto-resistive from 1990[17], and giant magneto resistive or tunneling magneto resistive heads from 1997[18, 17, 19] and 2004 onwards[18, 19, 17]. Servo and signal processing technologies have also advanced[20]. One option to further increase AD is to reduce the head-media-spacing, since this reduces the signal-to-noise ratio[21] and limits AD growth[21]. For this reason, the COC thickness reduced from 12.5nm in 1990[22], corresponding to 1Gb/in^2[22], to 2.5-3nm in 2013 for $0.8\text{-}1\text{Tb/in}^2$, with a planned reduction to 1.8-1.5nm for 4Tb/in^2 by 2021.

The main advantage of HDDs is that they can store a large amount of

data$\sim$TB cheaply. As of 2019, solid state drives (SSDs) are still more than twice more expensive than HDDs: A 1TB internal 2.5-inch HDD costs$\sim$\$40-50[23], while an SSD of the same capacity and form factor starts at$\sim$\$250[23]. The main advantage of SSDs over HDDs is that they do not have moving parts, making them more stable compared to HDDs. Another advantage is that SSDs based on NAND flash memories are faster than HDDs:$\sim$10-13s average boot-up time for SSDs[23], compared to$\sim$30-40s for HDDs[23].

In order to increase signal to noise ratio and AD, the grain size of the magnetic recording layer must be reduced[15, 12, 24, 25]. This requires an increase in the magnetic anisotropy of the grains, to ensure the magnetic state is stable (thermal stability). However, the magnetic field needed to switch the magnetic state depends on the anisotropy, and is larger for increasing anisotropy materials than what can be currently achieved in read and write heads (2T) (writability). In order to achieve higher AD the magnetic grain size would need to be reduced$<$8-9nm[15, 12]. However, this would result in thermal instability of the grains, due to decrease of their magnetic anisotropy energy[15, 31], whereby magnetization can randomly flip in response of thermal fluctuations[15]. This in turn requires an increase in the magnetic anisotropy of the grains to ensure the magnetic state is stable (thermal stability). For thermal stability of small grains$\sim$3nm over long periods of time (up to 10 years) the magnetic recording layer must have $\mathrm{K_u V/k_B T} \geq 40 - 60$[15, 26, 31], where K_u=1/2$H_K M_s$[15] is the anisotropy constant, H_K [A/m] is the magnetic anisotropy field, M_s [A/m] is the saturation magnetization, V [m^3] is the grain volume, k_B is the Boltzmann constant and T is the temperature. Thus, magnetic materials with large$>$100kOe H_K are needed to support grains down to 3nm. E.g., FePt supports stable grains with diameters down to$\sim$2-3nm[26, 31, 27, 28]. However, the magnetic field needed to switch the magnetic state depends on anisotropy, and is larger for high anisotropy materials than currently achieved in read and write heads (2T) (writability)[15, 26]. E.g., the FePt coercivity up to 50000Oe (10000Oe=1T)[26] is larger than in recording heads with$\sim$1-2T[15, 26].

To overcome the magnetic trilemma[29] and to be competitive with solid state drives[30], heat-assisted magnetic recording (HAMR) has been introduced. HAMR uses a laser to heat the magnetic medium for$\sim$1ns[31] in order to decrease its coercivity, i.e. the amount of reverse driving field required to demagnetize a magnetic medium[32], bringing the material to its Curie temperature[33].

Supplementary Note 2: Magnetic media substrate fabrication

The COC thickness reduced from 12.5nm in 1990, corresponding to 1Gb/in^2, to 2.5−3.0nm in 2013 for $0.8-1\text{Tb/in}^2$[25, 34], with a planned decrease to 1.8-1.5nm for 4Tb/in^2 by 2021[25].

The commercial COC is plasma-assisted chemical vapour deposition (PACVD) grown a-C:H followed by a nitrogen plasma surface treatment[34, 35, 36, 37, 38, 39, 40]. PACVD, magnetron sputtering and FCVA are commonly used to deposit COCs[41, 34, 42, 43, 44, 45, 46, 35, 36, 37, 38, 39, 47, 48, 40].

We also use magnetron sputtering[47] and FCVA to deposit and compare the performance of all types of COCs with 1-4LG. COCs with thicknesses from∼0.3 to∼1.8nm are deposited on Co-alloy-based HDM using pulsed DC magnetron sputtering[46, 52] and FCVA[41, 34, 44, 46, 35, 47, 48, 49, 50, 51]. A first COC set is deposited using the NTI Media coating system FS2 FCVA, equipped with a double bend macro-particles filter[35] at an ion energy∼50eV and dose$\sim 3.75\times10^{15}\text{ions/cm}^2$. The thickness is controlled by varying the deposition time, and the rate is calibrated using AFM and high resolution transmission electron microscopy (HRTEM)[35]. A second COC set is prepared using an AJA sputtering tool[46, 52]. Argon (Ar) plasma cleaning is performed for 3mins, prior to COC deposition, at RF power∼40W, Ar gas flow rate∼20sccm and pressure∼10mTorr to remove surface contamination[52]. 99.999% pure graphite is used as target. The COCs are grown in an Ar atmosphere with pressure and gas flow rate∼5mTorr and 20sccm. A pulsed DC power∼100W with 150kHz frequency and duration$\sim 2.6\mu$s is supplied to the graphite target. The deposition time is varied to achieve different thicknesses.

Supplementary Note 3: Lubricant coating

Graphene is an emerging material for lubrication[53, 54, 56, 57, 58, 55, 59, 60], as well as oxidation[61, 62] and corrosion protection[63, 64, 65].

Commercial HDM with COCs and commercial PFPE lube are sourced from Wester Digital, USA[40]. We also coat the other samples with PFPE Zdol 4000 as follows. The lube is dissolved in Vertrel XF. The samples are then immersed into and withdrawn from the PFPE solution at∼0.5mm/s. Before withdrawal, the samples are held for 20s to allow the lubricant molecules to adhere. The PFPE-coated samples are then cured at 150°C for 1.5h. This is done to improve the PFPE bonding to the surface[34]. The parameters are optimized to obtain a lubricant thickness$\sim 1.2\pm0.2$nm[34, 46, 40], similar to commercial HDM[40]. We also apply PFPE lube on BM (BML) to examine

the tribology of lube containing bare HDM.

Fourier transform infrared (FTIR) spectroscopy is employed to determine the lubricant thickness[66, 67]. The measurements are performed in reflectance mode and the absorption band$\sim$1280cm^{-1}, due to combination of C-F and C-O vibrations, is analyzed to estimate the thickness[40], and calibrated against the lubricant film thickness measured by X-ray photo electron spectroscopy[66, 67].

Supplementary Note 4: Auger electron spectroscopy

AES imaging is performed using a JEOL JAMP Auger Microprobe[68, 69]. The results are shown in Supplementary Fig.1.

Supplementary Note 5: Atomic force microscopy

The thickness and roughness of 1-4LG are measured by AFM. We use a Bruker Dimension Icon AFM in peak-force tapping mode.

The roughness of BM is$\sim$0.5nm, while that of BM+1-4LG is$\sim$0.6nm. This shows that there is no major change in roughness after 1-4LG transfer, and that the main contribution to the roughness comes from the BM substrate. Sp^3 rich amorphous carbon thin films are ultra-smooth with roughness$\sim$0.12nm[70, 71]. However, for such thin amorphous carbon films pin holes and island formation occur[70]. Therefore, graphene represents a suitable alternative.

In order to measure the thickness, we perform AFM measurements of 1LG transferred on Si/SiO_2. The step height is$\sim$0.7-1nm, Supplementary Fig.2. For 2LG this is$\sim$1.1-1.4nm, for 3LG$\sim$1.4-1.7nm, for 4LG$\sim$2-2.6nm, all below the commercial COC thickness. The topography resolution of a Bruker Dimension Icon in peak-force tapping mode is$\sim$50pm. It is well known that the height of 1L-layered materials (LMs) on a substrate does not reproduce the theoretical thickness, as we first discussed in Ref.[72]. This is related to different parameters: the roughness of the substrate; the force of interaction between 1L-LM and substrate; trapped contaminants. E.g. even for SLG, measured AFM thicknesses$\sim$1.8nm are common, depending on peak force and substrate[73].

We also perform AFM measurements on 1-4LG on HDM. To measure the step height of graphene transferred on HDM, we pattern it and take AFM measurements across the edge. The thickness of 4LG on HDM is$\sim$2.1nm, Supplementary Fig.3, well below the$\sim$2.7nm of commercial COC.

Supplementary Note 6: Magneto-optical Kerr effect measurements

Magneto-optical Kerr effect (MOKE) measurements are carried out on BM, BM+2-3nm COC, and BM+1-4LG. Polar Kerr effect hysteresis loops are acquired using a wide field Kerr microscope equipped with a computer controlled analyzer, and a polar electromagnet, swept in the range ±890mT. The samples are illuminated with a cross array of 8 white light emitting diodes of uniform intensity for polar Kerr sensitivity. A 20x microscope objective lens (NA 0.5) is used to image the sample surface with a 240mm^2 field of view using a CMOS camera, with 1920x1200 pixels, and 2x2 pixel binning. A$\sim$185μm diameter circular region of interest (corresponding to a diameter$\sim$300 pixels) is selected at the centre of the field of view, to be free of dust, and to avoid the edge of the field of view. A linear Faraday rotation due to the exposure of the objective lens to the polar field is removed by using a computer controlled analyzer to acquire loops with similar reflectivity at remanence, i.e. the residual magnetization after the external magnetic field is removed[74], but with opposite sign of the Faraday signal. First, the analyzer angle is calibrated by locating the minimum in its transmission when crossed with the incident polarization. The analyzer is then opened by$\sim 2-3°$ to provide a visible polar Kerr contrast, without saturating the pixels of the camera due to the Faraday effect, when swept over the full magnetic field range. A loop is then acquired at that analyzer angle, and the reflectivity noted at remanence. The analyzer is then opened to the other side of the crossed condition to an angle with equivalent reflectivity at remanence, and a second loop acquired. Since the Faraday effect has opposite sign in the two measurements, and the reflectivity is equal, subtraction of the loops eliminates the Faraday signal. The resulting doubling of the polar Kerr signal is corrected for by dividing the signal by 2.

To present a more accurate comparison between the MOKE loops measured on BM, BM+COC, BM+1-4LG, we normalize the MOKE hysteresis loops as follows. Normalization is carried out by dividing the measured Kerr intensity by a normalization factor $(I_{max} - I_{min})/2$, where I_{max} is the maximum and I_{min} is the minimum Kerr intensity of a measured loop. The results are plotted in Supplementary Fig.4 and agree well with MOKE measurements on CoCrPt:O alloys reported in literature[75].

Supplementary Fig.4 shows that coating results in a small change in the hysteresis loop. The coercivity changes from 510mT (BM) to$\sim$545mT (1-4LG and COC). The remanent magnetization is unchanged at$\sim$97-98% of the saturation magnetization compared to BM. Coercivity and remanent magnetization of 1-4LG coated BM are similar to BM+COC, indicating that

graphene coating does not have any detrimental effect on HDM. These provide a strong indication that the magnetisation in the recording layer is unchanged even after multiple graphene transfers.

The area enclosed by the loops corresponds to the energy stored, or the energy barrier for rotating the spins, and changes with size and number of magnetic domains[74]. For BM+COC this is∼4% higher than BM, while it is∼5-7% higher for BM+1-4LG, Supplementary Fig.5. The higher the energy barrier, the more difficult it is to switch from one state to the other (i.e. from up to down, or 0 to 1)[74]. So, an increase in energy indicates more stability of magnetization, with higher data retention, defined as the time the data is stable without degrading. MOKE data also indicate that there is no influence of moisture that might have been introduced on the media via graphene transfer, as the magnetic properties remain largely unchanged.

Supplementary Note 7: Razor-blade determination of laser spot size

We use the razor-blade technique to determine the laser spot size, as part of the irradiation stability measurements. This involves using a razor blade between objective and sample, such that it intersects the beam in a direction perpendicular to the propagation axis of the beam, and then moving it from fully covering the beam path until it does not cover the laser, while measuring the peak intensity[77]. If the profile of the beam is described by a Gaussian, the signal measured by the detector is represented by an integrated Gaussian[77], i.e. the derivative of the signal will give a Gaussian profile. We perform a line scan perpendicular to an Au contact, while measuring the Si peak intensity as function of scan position. We plot the Si peak intensity as a function of scan position. Taking the derivative of the intensity profile gives a Gaussian, and its $1/e^2$ width corresponds to the laser spot diameter[77]. This information is then used to analyse the laser irradiation stability of our CVD-grown graphene[76] on magnetic media. Refs.[78, 79] reported that suspended 1LG has good thermal stability up to 2600K, with a thermal conductivity up∼2000W/mK[78, 80, 81, 82, 83, 84].

Supplementary Note 8: Molecular dynamics simulations

Friction is defined as resistance to sliding[56, 85, 86]. At the macroscopic level, Amonton's law[85] states that the frictional force between two bodies varies proportionally to the normal force. The coefficient of friction, COF

is the ratio of frictional force to normal force[87, 88]. Since the layers in graphite are coupled by van-der-Waals forces, they shear easily[89, 90, 91], as their interfacial shear strength is low, with a shear force per unit area $\alpha \sim 12.8{\cdot}10^{18}$Nm^{-3}[92] and a resulting shear modulus C_{44} ~4.3GPa[92]. 1LG also shows excellent mechanical properties: breaking strength~42 Nm^{-1}[93], Young's Modulus~1TPa[93], flexibility (1LG can be stretched up to~20% without breaking[93, 94])

We use molecular dynamics (MD) simulations to study friction in graphene as described below.

The initial relaxation runs are carried out on a system with~10,000 atoms, periodic in X and Y. Simulations are performed in an isobaric (NPT [constant N- number of atoms, P- Pressure, T-temperature]) ensemble, with P controlled in the in-plane directions. A Nose-hoover barostat[95, 96] with time-constant=500fs is used. T is kept at 300K, using a Nose-Hoover thermostat with time-constant=50fs. The timestep is 0.5fs. Equilibration is performed for~1ns. The sliding runs are done in a microcanonical ensemble (NVE [constant N-number of atoms, V-volume, E-energy]) with a sliding velocity=1m/s. Sliding simulations are carried out for 600ps.

The MD simulations are performed using the reactive force field (ReaxFF) [97, 98] interatomic potential in order to capture the interactions between C in SLG and Al and O in sapphire. ReaxFF can describe chemical reactions and polarization, and enhances the physical realism of MD simulations[97, 98]. This is done by including variable charges and reactive bond orders that dynamically adapt to the local environment[100], at significant (i.e.~2-5 times higher than typical embedded atom models (EAMs)[100]) computational cost[100]. The potential parameters for C/Al/O interactions are taken from Refs.[101, 102]. The Co|Pt substrate is assumed to be a fixed wall, since the goal is to demonstrate that the structure of defected graphene, present as an interfacial layer between alumina and Co|Pt, does not undergo significant change due to friction at 300K, even with a sliding velocity of 1m/s. No chemical reactions occur between graphene and alumina. This indicates that it is sufficient to use non-reactive (i.e. with no chemical bond formation or breakage) van der Waals interactions to describe the cross-interactions between different elements. The cross-interaction across the Al_2O_3|SLG|Co-Pt interface is assumed to be van der Waals, and described by the 12-6 Lennard-Jones (LJ) potential using Lorentz-Berthelot rules. The interactions in graphene are described by the bond-order Tersoff interatomic potential[103].

The final MD simulations are performed in LAMMPS (Large-scale Atomic/ Molecular Massively Parallel Simulator)[104] with the hybrid/overlay pair style. Al_2O_3 is described by EAM parameters developed as part of the charge-transfer ionic potential (CTIP) formalism[105], which is an extension of the

Streitz-Mintmire interatomic potential[106]. The charges on Al and O are assumed to be fixed at 2.86 and -1.91, i.e. the charge equilibrated charges, Qeq, in bulk Al_2O_3 using the CTIP formalism[105].

No charge equilibration is performed during friction simulations, as we are interested in observing structural changes in SLG and Co|Pt due to friction. The interactions in SLG are described by the Tersoff potential of Ref.[103], while the Co|Pt substrate is described by the EAM potential of Ref.[107]. All cross-interactions are described via the LJ potential, with parameters obtained using Lorentz-Berthelot mixing rules[108]. The C-C, Pt-Pt, Al-Al, Co-Co, O-O LJ parameters used for mixing are in Supplementary Table 1.

The local disorder in the FCC Co|Pt substrate is identified using common neighbor analysis (CNA)[109, 110, 111] with adaptive/variable cutoff[111]. CNA is used to compute a fingerprint for atom pairs and to characterize the structural environment local to each atom. Such an approach is effective as a filtering method to classify atoms in crystalline systems[109, 110, 111]. We identify all non-FCC atoms as either surface atoms or bulk disorder. CNA also identifies surface atoms, which may not be disordered or defected[109, 110, 111]. In our simulations, we have two Co|Pt surfaces with 286 atoms each. Hence, we evaluate the number of disordered atoms in the bulk by subtracting the number 572 from the CNA output. The surface disorder is identified by evaluating the centrosymmetry parameter[112], which is a useful measure to characterize the local lattice disorder. The histogram of centrosymmetry parameters for the Co|Pt surface layer in the interfacial region is in Supplementary Fig.6.

A perfect, defect-free surface layer has atoms with 4 nearest-neighbors, used to evaluate the local environment of every atom. The inset of Supplementary Fig.6a shows the defect-free structure. From the histogram, a centrosymmetry parameter cutoff$\sim$1.5 is reasonable to identify defect atoms. Supplementary Fig.6b shows the histogram for a sample defected surface. The atoms in the inset of Supplementary Fig.6b are colored by the centrosymmetry parameter for values$\geq$1.5. The total number of disordered/defect atoms is NCS+NCNA$-$572, where NCS is the number of defect atoms on the surface identified by the centrosymmetry algorithm, and NCNA is the number of non-FCC atoms identified by the CNA algorithm. Supplementary Fig.7 shows how the surface defects change during frictional sliding at 1m/s of alumina on bare Co|Pt at 300K.

We use 350ps of simulation data for the initial centrosymmetry calculation, followed by an additional 250ps run, where the forces on atoms are also collected. Supplementary Fig.8 shows these additional 250ps (starting from 0). This is 250ps of more or less steady state. The sliding and normal force on a central group of atoms of substrate + interlayer are block averaged every

25ps. The friction coefficient in the MD simulations is estimated as the ratio of sliding and normal force, block averaged every 25ps, as for Ref.[113].

Supplementary Figs.9a-c compare the computed COF and surface disorder of Co|Pt, 1LG, 4LG. The surface disorder represents the friction induced damage/wear of the HDM, and its value is derived considering 0% surface disorder before friction measurements. The amount of disorder in HDM is quantified using the centrosymmetry parameter, which is a measure of the local lattice disorder around an atom[112]. This is 0 for a perfect lattice[112] whereas, when point defect exists, i.e. when symmetry is broken, it assumes a larger positive value[112].

Supplementary References

[1] Prutton, M. & Gomati, M. M. El. *Scanning Auger Electron Microscopy*, Eds., John Wiley and sons, (2006).

[2] Shen, M., Schelling, P. K., & Keblinski, P. Heat transfer mechanism across few-layer graphene by molecular dynamics. *Phys. Rev. B* **88**, 045444 (2013).

[3] Huang, S.-P., Mainardi, D. S. & Balbuena, P. B. Structure and dynamics of graphite-supported bimetallic nanoclusters. *Surface Science* **545**, 163-179 (2003).

[4] Agrawal, P. M., Rice, B. M. & Thompson, D. L. Predicting trends in rate parameters for self-diffusion on FCC metal surfaces. *Surface Science* **515**, 21-35 (2002).

[5] Rappe, A. K., Casewit, C. J., Colwell, K. S., Goddard III, W. A. & Skiff, W. M. UFF, a full periodic table force field for molecular mechanics and molecular dynamics simulations. *J. Am. Chem. Soc.* **114**, 10024 (1992).

[6] Harris, J. G. & Yung, K. H. Carbon diocide's liquid-vapor coexistence curve and critical properties as predicted by a simple molecular model *J. Phys. Chem.* **99**, 12021-12024 (1995).

[7] Granz, S. et al. Perpendicular Interlaced Magnetic Recording. *IEEE Trans. Magn.* **55**, 3100203 (2019).

[8] Hernandez, S. et al., Effect of recording conditions on the downtrack thermal gradient in Heat-Assisted Magnetic Recording. *IEEE Trans. Magn.* **54**, 1-4 (2018).

[9] Hwang, E., Park, J., Rauschmayer, R. & Wilson, B. Interlaced magnetic recording. *IEEE Trans. Magn.* **53**, 3101407 (2017).

[10] Grochowski, E. & Goglia, P. Magnetic hard disk drive today's technical status and future. `https://www.snia.org/sites/default/files/SDC/2016/presentations/keynote_general/Grochowski-Goglia_Magnetic_Hard_Disk_Drive.pdf`.

[11] WW HDD market declining to 395 million units shipped in 2017-statista. Storage Newsletter (2017). `https://www.storagenewsletter.com/2017/10/10/ww-hdd-market-declining-to-395-million-units/-shipped-in-2017-statista/`

[12] Piramanayagam, S. N. Perpendicular recording media for hard disk drives. *J. Appl. Phys.* **102**, 011301-22 (2007).

[13] McFadyen, I. R., Fullerton, E. E. & Carey, M. J. State-of-the art magnetic hard disk drives. *MRS Bull.* **31**, 379-383 (2006).

[14] Xiong, S. & Bogy, D. B. Hard disk drive servo system based on field-programmable gate arrays. *IEEE Transactions on Industrial Electronics* **61**, 4878 - 4884 (2014).

[15] Plumer, M. L., van Ek, J. & Cain, W. C. New Paradigms in Magnetic Recording. *Phys. Canada* **67**, 25-29 (2011).

[16] M. H. Kryder, Magnetic thin films for data storage. *Thin Solid Films* **216**, 174-180 (1992).

[17] Bajorek, C. H. *Magnetoresistive (MR) Heads and the Earliest MR Head-Based Disk Drives: Sawmill and Corsair.* Computer History Museum, Mountain View, CA. (2014)

[18] Mao, S. et al. Commercial TMR heads for hard disk drives: characterization and extendibility at 300 gbit^2. *IEEE Trans. Magn.* **42**, 97-102 (2006).

[19] Horowitz, R., Li, Y., Oldham, K., Kon, S. & Huang, X. Dual-stage servo systems and vibration compensation in computer hard disk drives. *Control Engineering Practice* **15**, 291-305 (2007).

[20] Zhang, T., Mathew, G., Zhong, H. & Micheloni, R. *Modern Hard Disk Drive Systems: Fundamental and Future Trends.* Memory mass storage, editors Campardo, G., Tiziani, F. & Iaculo, M. 169-212 (2010).

[21] Wallace, R. L. The Reproduction of Magnetically Recorded Signals. *Bell System Technology Journal* **30**, 1145-1173 (2000).

[22] Yogi, T., Nguyen, T. A., Lambert, S. E., Gorman, G. L. & Castillo, G. Role of atomic mobility in the transition noise of longitudinal media. *IEEE Transactions on Magnetics* **26**, 1578 - 1580 (1990).

[23] `https://www.storagereview.com/ssd_vs_hdd` (2018).

[24] Weller, D. et al. FePt heat assisted magnetic recording media. *J. Vac. Sci. Technol. B* **34**, 060801-10 (2016).

[25] Marchon, B. et al. The head-disk interface roadmap to an areal density of 4 Tbit/in^2. *Adv. Tribol.* **2013**, 521086 (2013).

[26] Plumer, M. L., van Ek, J. & Weller, D. *The Physics Of Ultrahigh Density Magnetic Recording.* Springer series in surface sciences, 1-352 (2001).

[27] Kryder, M. H. et al., Heat Assisted Magnetic Recording. *Proc. IEEE* **96**, 1810-1835 (2008).

[28] Challener, W. A. et al., Heat-assisted magnetic recording by a near-fieldtransducer with efficient optical energy transfer. *Nat. Photonics* **3**, 220-224 (2009).

[29] Richter, H. J. The transition from longitudinal to perpendicular recording. *J. Phys. D Appl. Phys.* **40**, R149-R177 (2007).

[30] `https://uk.crucial.com/gbr/en/learn-with-crucial/about-ssd/ssd-vs-hdd`, Micron Technolgies (2017).

[31] Weller, D. & Moser, A. Thermal effect limits in ultrahigh-density magnetic recording. *IEEE Trans. Magn.* **35**, 4423-4439 (1999).

[32] Hutchings, I. et al. *Springer Handbook of Materials Measurement Methods*, 685-710 (2006).

[33] Hu, Y., Wu, H., Meng, Y., & Bogy, D. B. Nanoscale thermal analysis for heat-assisted magnetic recording *J. Appl. Phys.* **122**, 134303 - 134303 (2017).

[34] Dwivedi, N. et al., Ultrathin carbon with interspersed graphene/fullerene-like nanostructures: a durable protective overcoat for high density magnetic storage. *Sci. Rep.* **5**, 11607 (2015).

[35] P. S. Goohpattader et al., Probing the role of C^+ ion energy, thickness and graded structure on the functional and microstructural characteristics of ultrathin carbon films (<2 nm). *Tribo. Int.* **81**, 73-88 (2015).

[36] Jones, P. M., Ahner, J., Platt, C. L., Tang, H. & Hohlfeld, J. Understanding Disk Carbon Loss Kinetics for Heat Assisted Magnetic Recording. *IEEE Trans. Magn.* **50**, 144-147 (2014).

[37] Ji, R., Ma, Y., Shakerzadeh, M., Seet, H. L. & Hu, J. F., Laser irradiation effect on carbon overcoat for HAMR application. *Surf. Interface Anal.* **46**, 204-208 (2014).

[38] Mangolini, F., Rose, F., Hilbert, J. & Carpick, R. W. Thermally induced evolution of hydrogenated amorphous carbon. *Appl. Phys. Lett.* **103**, 161605 - 161605-5 (2013).

[39] Wang, N., Komvopoulos, K., Rose, F. & Marchon, B. Structural stability of hydrogenated amorphous carbon overcoats used in heat-assisted magnetic recording investigated by rapid thermal annealing. *J. Appl. Phys.* **113**, 083517 (2013).

[40] Waltman, R. J. & Deng, H. Low Molecular Weight Z-Tetraol Boundary Lubricant Films in Hard Disk Drives. *Adv. Tribol.* **2012**, 964089 (2012).

[41] Casiraghi, C., Robertson, J. & Ferrari, A. C. Diamond-like carbon for data and beer storage. *Mater. Today* **10**, 44-53 (2007).

[42] Dwivedi, N., Yeo, R. J., Satyanarayana, N., Kundu, S., Tripathy, S. & Bhatia, C. S. Understanding the role of nitrogen in plasma-assisted surface modification of magnetic recording media with and without ultrathin carbon overcoats. *Sci. Rep.* **5**, 7772 (2015).

[43] Rajauria, S., Canchi, S. V., Schreck, E. & Marchon, B. Nanoscale wear and kinetic friction between atomically smooth surfaces sliding at high speeds. *Appl. Phys. Lett.* **106**, 081604 (2015).

[44] Ferrari, A. C. Diamond-like carbon for magnetic storage disks. *Diam. Rel. Mats.* **180-181**, 190-206 (2004).

[45] Casiraghi, C. et al. Ultra-thin carbon layer for high density magnetic storage devices. *Diam. Rel. Mats.* **13**, 1480-1485 (2004).

[46] N. Dwivedi et al., Atomic scale interface manipulation, structural engineering, and their impact on ultrathin carbon films in controlling wear,

friction, and corrosion. *ACS Appl. Mater. Interfaces* **8**, 17606-17621 (2016).

[47] Kundu, S. et al. Probing the role of carbon microstructure on the thermal stability and performance of ultrathin (<2 nm) overcoats on L10 FePt media for Heat-Assisted Magnetic Recording. *Mater. Interfaces* **7**, 158-165 (2015).

[48] B. K. Pathem et al., Carbon overcoat oxidation in heat-assisted magnetic recording. *IEEE Trans. Magn.* **49**, 3721-3724 (2014).

[49] Ferrari, A. C., Kleinsorge, B., Morrison, N. A. , Hart, A., Stolojan, V. & Robertson, J. Stress reduction and bond stability during thermal annealing of tetrahedral amorphous carbon. *J. Appl. Phys.* **85**, 7191-7197 (1999).

[50] Polo, M. C., Andujar, J. L., Hart, A., Robertson, J. & Milne, W. I. Preparation of tetrahedral amorphous carbon films by filtered cathodic vacuum arc deposition. *Diamond Rel. Mat.* **9**, 663-667 (2000).

[51] Fallon, P. J., Veerasamy, V. S., Davis, C. A., Robertson, J., Amaratunga, G. A. J. , & Milne, W. I. Properties of filtered-ion-beam-deposited diamondlike carbon as a function of ion energy. *Phys. Rev. B* **48**, 4777-4782 (1993).

[52] Dwivedi, N., Yeo, R. J., Goohpattader, P. S., Satyanarayana, N., Tripathy, S. & Bhatia, C. S. Enhanced characteristics of pulsed DC sputtered ultrathin (< 2 nm) amorphous carbon overcoats on hard disk magnetic media. *Diam. Relat. Mater.* **51**, 14-23 (2015).

[53] Marchetto, D., Held, C., Hausen, F., Wahlisch, F., Dienwiebel, M.& Bennewitz, R. Friction and Wear on Single-Layer Epitaxial Graphene in Multi-Asperity Contacts. *Tribol. Lett.* **48**, 77-82 (2012).

[54] Berman, D., Deshmukh, S. A., Subramanian, S. K. R. S., Erdemir, A. & Sumant, A. V. Friction. Macroscale superlubricity enabled by graphene nanoscroll formation. *Science* **348**, 1118-1122 (2015).

[55] Berman, D., Erdemir, A. & Sumant, A. V. Reduced wear and friction enabled by graphene layers on sliding steel surfaces in dry nitrogen. *Carbon* **59**, 167-175 (2013).

[56] Berman, D., Erdemir, A. & Sumant, A. V. Graphene: a new emerging lubricant. *Mater. Today* **17**, 31-42 (2014).

[57] Berman, D., Deshmukh, S. A., Subramanian, S. K. R. S., Erdemir, A. & Sumant, A. V. Extraordinary Macroscale Wear Resistance of One Atom Thick Graphene Layer. *Adv. Funct. Mater.* **24**, 6640-6646 (2014).

[58] Berman, D., Erdemir, A. & Sumant, A. V. Graphene as a protective coating and superior lubricant for electrical contacts. *Appl. Phys. Lett.* **105**, 231907-4 (2014).

[59] C. Lee et al., Frictional characteristics of atomically thin sheets. *Science* **328**, 76-80 (2010).

[60] Egberts, P., Han, G. H., Liu, X. Z., Johnson, A. T. C. & Carpick, R. W. Frictional behavior of atomically thin sheets: hexagonal-shaped graphene islands grown on copper by chemical vapor deposition. *ACS Nano* **8**, 5010-5021 (2014).

[61] Topsakal, M., Sahin, H. & Ciraci, S. Graphene coatings: An efficient protection from oxidation. *Phys. Rev. B* **85**, 155445 (2012).

[62] Chen, S. et al. Oxidation Resistance of Graphene-Coated Cu and Cu/Ni Alloy. *ACS Nano* **5**, 1321-1327 (2011).

[63] M. B. Martin et al., Protecting nickel with graphene spin-filtering membranes: A single layer is enough. *Appl. Phys. Lett.* **107**, 012408-4 (2015).

[64] Prasai, D., Tuberquia, J. C., Harl, R. R., Jennings, G. K. & Bolotin, K. I. Graphene: corrosion-inhibiting coating. *ACS Nano* **6**, 1102-1108 (2012).

[65] Raman, R. K. S. et al., Protecting copper from electrochemical degradation by graphene coating. *Carbon* **50**, 4040-4045 (2012).

[66] Waltman, R. J., Kobayashi, N., Shirai, K., Khurshudov, A. &. Deng, H. The tribological properties of a new cyclotriphosphazene-terminated perfluoropolyether lubricant. *Tribology Letters* **16**, 151-162 (2004).

[67] Abdul Samad, M., Yang, H., Sinha, S. K. & Bhatia, C. S. Effect of carbon embedding on the tribological properties of magnetic media surface with and without a perfluoropolyether (PFPE) layer. *Journal of Physics D: Applied Physics*, **44**, 315301 (2011).

[68] Watts, J. F. & Wolstenholme, J. *An Introduction to Surface Analysis by XPS and AES*, Wiley, Chichester, (2003).

[69] Briggs, D. & Grant, J. T. (eds). *Surface Analysis by Auger and Xray Photoelectron Spectroscopy*, IMPublications, Chichester, UK and Surface Spectra, Manchester, UK, (2003)

[70] Casiraghi, C., Ferrari, A. C., Ohr, R., Flewitt, A. J., Chu, D. P. & Robertson, J. Dynamic Roughening of Tetrahedral Amorphous Carbon. *Phys. Rev. Lett.* **91**, 226104 (2003).

[71] Moseler, M., Gumbsch, P., Casiraghi, C., Ferrari, A. C. & Robertson, J. The ultrasmoothness of diamond-like carbon surfaces. *Science* **309**, 1545-1548 (2005);

[72] Ferrari, A. C. et al. Raman Spectrum of Graphene and Graphene Layers. *Phys. Rev. Lett.* **97**, 187401 (2006).

[73] Shearer, C. J., Slattery, A. D., Stapleton, A. J., Shapter, J. G. & Gibson, C. T. Accurate thickness measurement of graphene. *Nanotechnology* **27**, 125704 (2016).

[74] O'Handley, Robert C. *Modern Magnetic Materials*, Wiley, (2000).

[75] Navas, D. et al. Microscopic reversal magnetization mechanisms in CoCrPt thin films with perpendicular magnetic anisotropy: Fractal structure versus labyrinth stripe domains. *Phys. Rev. B.* **96**, 180403(R) (2017).

[76] Bae, S et al., Roll-to-roll production of 30-inch graphene filmsfor transparent electrodes. *Nat. Nanotechnol.* **5**, 574-578 (2010).

[77] Khosrofian, J. M. & Garetz, B. A., Measurement of a Gaussian laser beam diameter through the direct inversion of knife-edge data. *Appl. Opt.* **22**, 3406-3410 (1983).

[78] Yu, P., Yu, S. & Zhou, W. Evaluation of thermal performance of graphene overcoat on multi-layered structure subject to laser heating. *Int. J. Heat Mass Trans.* **68**, 27-31 (2015).

[79] Kim, K. et al. High-temperature stability of suspended single-layer graphene. *Phys. Stat. Solidi RRL* **4**, 302-304 (2010).

[80] Lee, J. U., Yoon, D., Kim, H., Lee, S. W. & Cheong, H. Thermal conductivity of suspended pristine graphene measured by Raman spectroscopy. *Phys. Rev. B* **83**, 081419 (2011).

[81] Faugeras, C., Faugeras, B., Orlita, M., Potemski, M., Nair, R. R. & Geim, A. K. Thermal conductivity of graphene in corbino membrane geometry. *ACS Nano* **4**, 18891892 (2010)

[82] Seol, J. H. et al. Two-dimensional phonon transport in supported graphene. *Science* **328**, 213-216 (2010).

[83] Cai, W. et al. Thermal transport in suspended and supported monolayer graphene grown by chemical vapor deposition. *Nano Lett.* **10**, 16451651 (2010).

[84] Chen, S. et al. Raman measurements of thermal transport in suspended monolayer graphene of variable sizes in vacuum and gaseous environments. *ACS Nano* **5**, 321-8 (2011).

[85] Postnikov, S. N. The friction of metals, *Contemp. Phys.* **6**, 94-111 (1964).

[86] Krim, J. Resource Letter: FMMLS-1: Friction at macroscopic and microscopic length scales. *Am. J. Phys.* **70**, 890 (2002).

[87] Bowden, F. P. & Tabor, D. *Friction: An Introduction To Tribology*, Anchor Press (1973)

[88] Bowden, F. P. & Tabor, D. *The Friction and Lubrication of Solids*, Oxford University Press, Oxford Classic Texts in the Physical Sciences, reprint edition (2001).

[89] Daly, M., Cao, C., Sun, H., Sun, Y., Filleter, T. & Singh, C. V. Interfacial shear strength of multilayer graphene oxide films, *ACS Nano* **10**, 1939-1947 (2016).

[90] Wang, G. et al. Measuring interlayer shear stress in bilayer graphene. *Phys. Rev. Lett.* **119**, 036101 (2017).

[91] Ruiz, L., Xia, W., Meng, Z. & Keten, S. A coarse-grained model for the mechanical behavior of multi-layer graphene. *Carbon* **82**, 103-115 (2015).

[92] P. H. Tan et al., The shear mode of multilayer graphene. *Nature Materials* **11**, 294-300 (2012).

[93] Lee, C., Wei, X. D., Kysar, J. W., Hone, J. Measurement of the elastic properties and intrinsic strength of monolayer graphene. *Science* **321**, 385-388 (2008).

[94] Bunch, J. S. et al. Electromechanical resonators from graphene sheets. *Science* **315**, 490-493 (2007).

[95] Nosé, S. A unified formulation of the constant temperature molecular dynamics methods. *J. Chem. Phys.* **81**, 511-519 (1984).

[96] Hoover, W. G. Canonical dynamics: Equilibrium phase-space distributions. *Phys. Rev. A* **31**, 1695 (1085).

[97] van Duin, A. C. T., Dasgupta, S., Lorant, F. & Goddard III, W. A. ReaxFF: A Reactive Force Field for Hydrocarbons. *J. Phys. Chem. A*, **105**, 9396-9409 (2001).

[98] Zhang, Q., Cagin, T., van Duin, A. C. T., Goddard III, W. A., Qi, Y. & Hector Jr., L.G. Adhesion and nonwetting wetting transition in the Al/α-Al_2O_3 interface. *Phys. Rev. B* **69**, 045423 (2004).

[99] Stansbury, E. E. & Buchanan, R. A. *Fundamentals of Electrochemical Corrosion*, ASM International (2000).

[100] Vashista, P., Kalia, R. K. & Nakano, A. Multimillion atom simulations of dynamics of oxidation of an aluminum nanoparticle and nanoindentation on ceramics. *J. Phys. Chem. B* **110**, 3727-3733 (2006).

[101] Sen, F. G. , Qi, Y., van Duin, A. C. T. & Alpas, A. T. Oxidation induced softening in Al nanowires. *Appl. Phys. Lett.* **102**, 051912 (2013)

[102] Sen, F. G., Alpas, A. T., van Duin, A. C. T. & Qi, Y. Oxidation-assisted ductility of aluminium nanowires. *Nat. Comms.* **5**, 3959 (2014).

[103] Lindsay, L. & Broido, D. A. Optimized Tersoff and Brenner empirical potential parameters for lattice dynamics and phonon thermal transport in carbon nanotubes and graphene. *Phys. Rev. B* **81**, 205441 (2010).

[104] Plimpton, S. Fast Parallel Algorithms for Short-Range Molecular Dynamics. *J. Comp. Phys.* **117**, 1 (1995).

[105] Zhou, X. W., Wadley, H. N. G., Filhol, J.-S. & Neurock, M. N. Modified charge transferembedded atom method potential for metal/metal oxide systems. *Phys. Rev. B* **69**, 035402 (2004).

[106] Streitz, F. H.& Mintmire, J. W. Electrostatic potentials for metal-oxide surfaces and interfaces. *Phys. Rev. B* **50**, 11996 (1994).

[107] Zhou, X. W., Johnson, R. A.& Wadley, H. N. G. Misfit-energy-increasing dislocations in vapor-deposited CoFe/NiFe multilayers. *Phys. Rev. B* **69**, 144113 (2004).

[108] Allen, M. P. & Tildesley, D. J. *Computer Simulation of Liquids*, Oxford University Press, Oxford (1990).

[109] Honeycutt, J. D. & Andersen, H .C. Molecular dynamics study of melting adn freezing of small Lennard-Jones clusters. *J. Phys. Chem.* **91**, 4950-4963 (1987).

[110] Faken, D. & Jonsson, H. Systematic analysis of local atomic structure combined with 3D computer graphics. *Computational Materials Science* **2**, 279-286 (1994).

[111] A. Stukowsi, Structure identification methods for atomistic simulations of crystalline materials. *Modelling and Simulation in Materials Science and Engineering* **20**, 045021 (2012).

[112] Kelchner, C. L., Plimpton, S. J. & Hamilton, J. C. Dislocation nucleation and defect structure during surface indentation. *Phys. Rev. B* **58**, 11085 (1998).

[113] Gao, G., Cannara, R. J., Carpick, R. W. & Harrison, J. A. Atomic-scale friction on diamond: A comparison of different sliding direction on (001) and (111) surfaces using MD and AFM. *Langmuir* **23**, 53945405 (2007).
